# Chromatographic purification of the plasmin‐like enzyme from clamworm (*Perinereis aibuhitensis* Grub) and its fibrinolytic activity by metal ions

**DOI:** 10.1096/fj.202400086RR

**Published:** 2024-06-26

**Authors:** Tuo Song, Xinyuan Han, Tingting Jiang, Xinyi Pu, Mingjun Wei, Yuping Zhu, Wenhui Wu

**Affiliations:** ^1^ Department of Marine Biopharmacology, College of Food Science and Technology Shanghai Ocean University Shanghai China; ^2^ East China Sea Marine Biological Resources Engineering Technology Center Changzhou China; ^3^ Innovative Platform for Marine Biopharmaceutical Technology in Lingang New Area Shanghai China; ^4^ Department of Clinical Medicine Naval Medical University Shanghai China

**Keywords:** fibrinolytic activity measure, fibrinolytic protease, *Perinereis aibuhitensis* Grub, protease purification

## Abstract

In this study, fibrinolytic protease was isolated and purified from *Perinereis aibuhitensis* Grub, and the extraction process was optimized. The properties of the enzyme, such as the amino acid composition, thermal stability, optimal temperature, and pH, were investigated. After detoxification, proteins collected from fresh Clamworm (*Perinereis aibuhitensis* Grub) were concentrated via ammonium sulfate precipitation. The crude protease was purified using gel filtration resin (Sephadex G‐100), anion exchange resin (DEAE‐Sepharose FF), and hydrophobic resin (Phenyl Sepharose 6FF). The molecular weight of the protease was determined by polyacrylamide gel electrophoresis (SDS‐PAGE). The optimum temperature and optimum pH of the protease were determined. The activity of crude protease in the 40–60% salt‐out section was the highest, reaching 467.53 U/mg. The optimal process for purifying crude protein involved the application of DEAE‐Sepharose FF and Phenyl Sepharose 6FF, which resulted in the isolation of a single protease known as Asp60‐D1‐P1 with the highest fibrinolytic activity; additionally, the enzyme activity was measured at 3367.76 U/mg. Analysis by Native‐PAGE and SDS‐PAGE revealed that the molecular weight of Asp60‐D1‐P1 was 44.5 kDa, which consisted of two subunits with molecular weights of 6.5 and 37.8 kDa, respectively. The optimum temperature for Asp60‐D1‐P1 was 40°C, and the optimal pH was 8.0.

## Graphical abstract

In this study, fibrinolytic protease was isolated and purified from *Perinereis aibuhitensis* Grub, and the extraction process was optimized. The properties of the enzyme, such as the amino acid composition, thermal stability, optimal temperature, and pH, were investigated. the isolation of a single protease known as Asp60‐D1‐P1 with the highest fibrinolytic activity; additionally, the enzyme activity was measured at 3367.76 U/mg. Analysis by Native‐PAGE and SDS‐PAGE revealed that the molecular weight of Asp60‐D1‐P1 was 44.5 kDa.

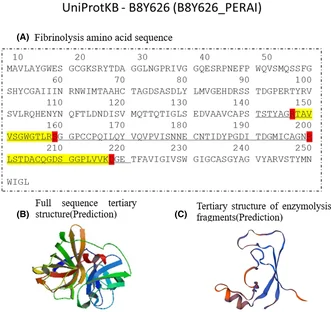

## 1 INTRODUCTION

Clamworm (*Perinereis aibuhitensis* Grub) is a kind of Nereid that is commonly found in the intertidal zones abundant in organic sediments along the southeast coast of China.^1^ The protein content in the clamworm can be as high as 60%. Clamworm protein contains 18 kinds of amino acids that are essential for humans. Additionally, the essential amino acid content (EAA/TAA) and the composition ratio (EAA/NAA) are approximately 38% and 69%, respectively, which is close to the protein ideal model recommended by the WHO (EAA/TAA ≥ 40%, EAA/NAA ≥ 60%); these values indicate high‐quality proteins that have a high nutritional value.^2^ The total fat content of clamworms is low (~10%), but unsaturated fatty acids constitute over 62% of the total fatty acids. The proportion of ω3 and ω6 polyunsaturated fatty acids can reach up to 43% and 57% of the total fatty acid content in the mature Clamworm.^3^ With the rapid advancement of the exploration of macromolecular compounds from marine resources, studies on clamworms have primarily focused on investigating their bioactive peptides. Several proteins from clamworms with pharmaceutical potential have been discovered, including those with anti‐cancer,^4^ anti‐thrombotic,^5^ anti‐hypertensive,^6^ anti‐microbial,^7^ and neurotransmitter^8^ activities.

Cardiovascular disease (CVD) is a leading cause of human death in modern society. About 290 million patients are currently suffering from cardiovascular diseases in China; among them, about 270 million are hypertensive.^9^ Acute myocardial infarction (AMI) is the leading cause of death among patients with CVD, the primary reason being a continuous increase in the standardized mortality rate since 2005; cases of AMI reached 64.12 per 100 000 in 2016, although the mortality rate of AMI in some developed countries in Europe and the United States has decreased over the years.^10^ AMI is mainly caused by the rupture of coronary atherosclerotic plaque, resulting in coronary artery obstruction, which in turn leads to myocardial ischemia and subsequent local myocardial necrosis. Within 12 h of the onset of AMI, the mortality rate increases by 1.6 per thousand for each additional hour of thrombolysis time. Therefore, the D‐to‐B time can be effectively controlled and shortened. The recanalization time is very important for treating AMI patients.^11^ Thrombolytics can be administered orally or intravenously after the onset of AMI to quickly degrade the fibrin in the thrombus, thus promoting the rupture of hardened plaques, which facilitates cardiac blood flow reperfusion in a short time, effectively prolongs the D‐to‐B time, and improves the treatment rate of patients. Therefore, the development of thrombolytic agents and their precursor active substances is a topic of active research in the field of CVD.

The study of clamworm with high nutritional value is not limited to ecology and aquaculture. Some researchers have extracted active antithrombotic peptides from the tissue of Clamworm. In 2001, Chen Hao et al.^12^ extracted fibrinolytic protease from *Peridentata bidentata* for the first time by gel filtration chromatography. In 2007, Wang Jingchao et al.^13^ obtained a protease with fibrinolytic activity composed of two subunits through three‐step purification by gel filtration chromatography, anion exchange chromatography, and cation exchange chromatography. They found that the optimal pH was 8–10 and the optimal temperature was 20–35°C. Zhang Yunlong et al.^14^ purified the liquid from the body cavity of *Bombyx* larva by four steps of anion exchange chromatography, weak cation exchange chromatography, and two gel filtration chromatography. In 2008, Li Qi et al.^15^ purified three fibrinolytic active proteases composed of polypeptide chains from *Bomboma japonica* through two steps of gel filtration chromatography and hydrophobic chromatography.

In 2007, Wang et al. used chromatography to purify a protease with fibrinolytic effect from clamworms. The molecular weight of the purified protease was 66 kDa. The optimal pH was 8–10, and the optimal temperature was 20–35°C.^16^ Zhang et al. also used chromatography to purify a single‐chain protease with strong fibrinolytic activity from Clamworm; its molecular weight was 29 kDa, and its isoelectric point was 4.5.^1^, ^17^ In 2008, Li et al. obtained three polypeptide chains with molecular weights of 29.3 kDa (a), 29 kDa (b), and 28.9 kDa (c) from the Clamworm through a two‐step chromatography purification process. They found that its optimal pH was 8–9, and the optimal temperature was 40–50°C. Polypeptides b and c have immunological homology. A rat platelet mutagenesis model showed that this enzyme can also significantly inhibit platelet aggregation.^18^ In 2016, Cao used hydrophobic chromatography, anion exchange chromatography, and hydrophobic chromatography to extract the fibrinolytic active protease from clamworm and found that its optimum temperature was 50°C, optimum pH was 8.0, and molecular weight was 32.5 kDa.^19^ However, due to the differences in purification and research methods, the results related to the molecular weight, isoelectric point, and basic physical and chemical properties of the fibrinolytic active protease of the bidentate silkworm are not clear.

In this study, *Perinereis aibuhitensis* Grub was used to conduct a systematic analysis of different chromatography purification methods and determine the best purification method. Additionally, the fundamental physical and chemical properties of the purified fibrinolytic active protease and the structure of polypeptide fragments were determined and simulated, which laid the foundation for investigating the follow‐up clamworm fibrinolytic protease.

## 2 MATERIALS AND METHODS

### 2.1 Materials and reagents

The Clamworm (*Perinereis aibuhitensis* Grub) was purchased from Yancheng Fengyueyuan Biotechnology Co., Ltd. in May 2018 and was transported to the laboratory in vivo under controlled temperature conditions (4°C).

The chemical reagents used were as follows. Sodium citrate, sodium chloride, trichloroacetic acid, casein, and Folin phenol reagent were purchased from Sangong Bioengineering (Shanghai) Co., Ltd., Shanghai. Potassium chloride, calcium chloride, magnesium chloride, ferric chloride, ferrous chloride, and concentrated hydrochloric acid were purchased from China National Pharmaceutical Reagent Co., Ltd., Shanghai. Coomassie Brilliant Blue R‐250 stain and normal saline were purchased from Biyuntian Biotechnology Co., Ltd., Nanjing. Triethylamine acetonitrile, phenyl isothiocyanate acetonitrile, anhydrous sodium acetate, glacial acetic acid, acetonitrile, and methanol were purchased from Sangong Bioengineering (Shanghai) Co., Ltd., Shanghai.

The biological reagents used were as follows. Bovine serum albumin (BSA), BCA kit, and agarose were purchased from Bi Yuntian Biotechnology Co., Ltd., Nanjing. Standard human plasmin freeze‐dried powder (500 UG, ≥2.0 units/mg), fibrinogen, and thrombin were purchased from Sigma, USA. Protein Marker (15 KDa‐100 KDa), 5× non‐denatured/denatured protein loading buffer, and 10× non‐denatured/denatured electrode buffer were purchased from Biyuntian Biotechnology Co., Ltd., Nanjing.

The chromatographic materials used were as follows. Sephadex G‐100 (Sephadex G‐100), DEAE‐Sepharose Fast Flow (DEAE‐Sepharose Fast Flow), and Phenyl‐Sepharose Fast Flow (Phenyl)‐Sepharose Fast Flow.

Consumables: 10 KDa dialysis bag (Solarbio Biotechnology Co., Ltd., Beijing); Non‐denaturing electrophoresis kit and denaturing electrophoresis kit (Sangong Bioengineering (Shanghai) Co., Ltd., Shanghai).

### 2.2 Systematic purification process design

#### 2.2.1 Crude protease extraction

The active clamworm was washed with clean water until no sediment residue was present on the body. The live clamworm was placed in a 1% citrate sodium chloride solution to hydrolyze the shell, after which, the intestinal sediment was removed, and the sandworm toxins were removed and inactivated.^20^ The inactivated clamworm was washed with distilled water. After draining the water, a one‐fold volume of PBS buffer (50 mM, pH 7.4) was added to homogenize the clamworm (4°C), followed by centrifugation for 15 min (4°C, 10 000 rpm) to obtain the supernatant. The precipitate was washed with 0.5 times the volume of PBS (20 mM, pH 7.4) and centrifuged under refrigeration. The supernatant was combined, and the step was repeated twice. The supernatant contained the crude extract of the protease from the clamworm.

Ammonium sulfate was added to the crude protease extract, and the ammonium sulfate saturation of the crude clamworm protease extract was adjusted to 40%, 60%, and 80%. After stirring for 15 min on a magnetic stirrer, it was centrifuged at high speed (4°C, 8000 rpm, 10 min) to collect the precipitate, which contained the salted‐out concentrated protein. The entire salting‐out process was kept at a low temperature (~4°C). The protein concentrates were placed at the above three salting out saturations in a 10 KDa dialysis bag. After dialysis was performed in double distilled water at 4°C for 24 h to remove the salt, the dialyzed protease solution was aliquoted into a Petri dish and frozen at −80°C for 12 h and then freeze‐dried with a lyophilizer for 24 h until the free water was completely sublimated. The dried, lyophilized sample contained the crude protein enzyme from the silkworm protein. The crude protein enzyme under the degree of salting out of 40%, 60%, and 80% was recorded as Asp40, Asp60, and Asp80, respectively.

#### 2.2.2 Chromatographic setting

In this study, three types of chromatography were performed, including gel filtration chromatography (Sephadex G‐100), anion exchange chromatography (DEAE‐Sepharose FF), and hydrophobic chromatography (Phenyl‐Sepharose FF). The specifications are as follows:

Sephadex G‐100 chromatography column (11 cm × 4.5 cm × 4.5 cm): Equilibration buffer and elution buffer were both 0.02 M Tris–HCl (pH 7.4), and the SephadexG‐100 filler was used in equilibration buffer. After activation at 4°C for 1 h, slowly add the resin to the column. The column was equilibrated with a flow rate of 3 mL/min with the equilibration buffer, and air bubbles were eliminated. After the baseline level stabilized, the protease solution was loaded.

DEAE‐Sepharose FF chromatography column (9 cm × 3.5 cm × 3.5 cm): the equilibration buffer used was 0.02 M Tris–HCl (pH 7.4), the elution buffer used was 0.02 M Tris–HCl (pH 7.4) + 1 M NaCl. The DEAE‐Sepharose FF packing material was placed with an equilibration buffer at 4°C for 1 h after activation and then filled in the column. The column was equilibrated with the equilibration buffer at a flow rate of 3 mL/min until the baseline was stable, and then, the protease solution was loaded.

Phenyl‐Sepharose 6FF chromatography column (8 cm × 3.5 cm × 3.5 cm): the equilibration buffer used was 0.05 M PBS (pH 7.4), the elution buffer was 0.05 M PBS (pH 7.4) + 1.7 M (NH_4_)_2_SO_4_, The Phenyl‐Sepharose FF packing was placed with the equilibration buffer at 4°C for 1 h, and then, it was filled in the column. The equilibration buffer was used to flush the column at a flow rate of 3 mL/min until the baseline was stable. Finally, the protease solution was loaded.

#### 2.2.3 Chromatography sequence design

Three chromatograms, including Sephadex G‐100, DEAE‐Sepharose FF, and Phenyl‐Sepharose FF, were used to fractionate the crude protease in different order. According to the protease activity and fibrinolytic activity after each step of chromatography, the best way to purify the fibrinolytic active protease of the larvae was determined.

### 2.3 Fibrinolytic activity assays

#### 2.3.1 Agarose‐fibrin plate method (qualitative)

Following the method described by Astrup,^21^ first, 5 mL of 1 mg/mL fibrinogen and 1 mL of 25 U/mL thrombin solution were placed in a 37°C constant temperature incubator and incubated for 30 min. The incubated thrombin was added to the Petri dish and shaken slightly to make it evenly distributed in the Petri dish. When the agarose solution was cooled to 60°C, 10 mL of 2% agarose solution and 5 mL of 1 mg/mL fibrinogen solution were mixed well. The plate containing thrombin was quickly added; the plate was slightly shaken to evenly mix the fibrinogen and thrombin at 37°C, and then, left undisturbed for 1 h to fully activate fibrinogen into fibrin by thrombin. Next, the mixture was placed in an oven at 85°C for 15 min to inactivate the plasminogen that might be present in the plate. The fibrin plate was taken out and placed at room temperature for about 1 h to stabilize.

The fibrin plate was punched with a sterile fine‐hole glue‐tip dropper, and 10 μL of 50 mg/mL protease solution was added to each hole. The plate was placed in a 37°C constant temperature incubator for 18 h. After incubation, it was stained with Coomassie Brilliant Blue R‐250 stain for 30 min, followed by decolorization with a decolorizing solution (acetic acid: methanol: ddH2O = 1:2:7) until the transparent circle was clear. The fibrinolytic activity of the purified protease was qualitatively analyzed according to the size of the transparent circle. The UVP system was used to record the results of the fiber slab.

#### 2.3.2 AUV spectrophotometry method (quantitative)

Following the method described by Wei et al.^22^ and according to the fibrinolytic cascade, the fibrinolytic activity was quantified by observing the size of the hydrolysis transparent circle of protease for a certain period.

Plotting the FDP standard curve: Five 0.6 mL centrifuge tubes were numbered, after which 70 μL of PBS buffer, 70 μL of fibrinogen solution, and 10 μL of thrombin solution were added to the centrifuge tubes. The mixture was incubated at 37°C for 10 min to generate fibrin, and then, it was placed in each tube. Next, 10 μL of standard human plasmin solution was mixed with 0.6, 0.8, 1.0, 1.2, and 1.4 mU/μL, followed by incubation at 37°C for 20 min. Immediately after incubation, 70 μL of TCA was added to stop the reaction. After centrifuging the mixture for 5 min (12 000 rpm, 4°C), 100 μL of supernatant was added from each tube to the UV 96‐well microtiter plate. Finally, the absorbance was measured at 273 nm, and the fibrinolytic activity standard curve was plotted (Figure 1).

**FIGURE 1:**
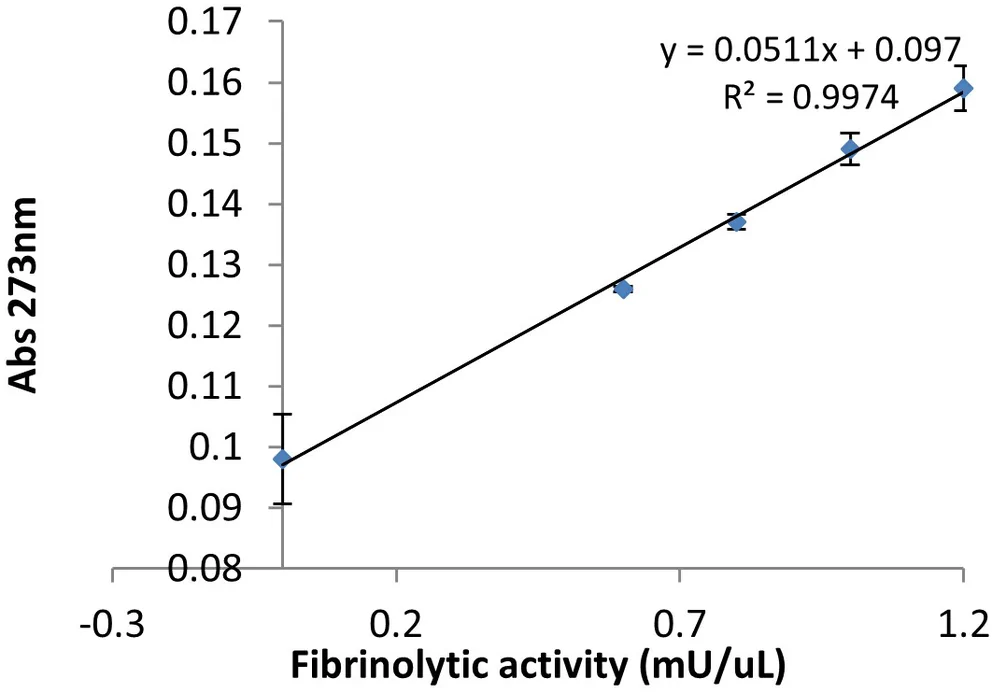
The standard curve of protein quantity was determined by the BCA method.

First, 2.0 ± 0.1 mg of freeze‐dried powder sample was dissolved in 40 μL of physiological saline to prepare a 50 mg/mL sample solution. After incubating 70 μL of PBS, 70 μL of fibrinogen solution, and 10 μL of thrombin at 37°C for 30 min, 10 μL of the prepared sample solution was immediately added at 37°C and allowed to react for 20 min. Then, 70 μL of TCA was added, and the mixture was left undisturbed for 5 min to stop the reaction, after which, it was centrifuged (12 000 rpm, 4°C) and 100 μL of the supernatant was collected. The absorbance was measured at 273 nm in an ultraviolet 96‐well microtiter plate, and the fibrinolytic activity of the sample was calculated according to the standard curve (the fibrinolytic activity of protease was defined as the amount of fibrin hydrolysis of 1 μg of protein per minute):

$\mathrm{fA}=\frac{\mathrm{Ab}}{K}\times \frac{230}{10}\times \frac{1}{W}\times N$ (fA: fibrinolytic activity in mU/μg; Ab: sample absorbance at 273 nm; K: fibrinolytic activity standard curve slope; 230/10: 230 μL of the reaction system contains 10 μL of sample; N: sample dilution factor; W: lyophilized powder sample mass in mg).

### 2.4 Protease activity assay (Lowry method)

The enzyme activity of the protease was determined by the Folin‐phenol method (Lowry method).^23^

After placing tyrosine in an oven (75°C) for 2 h at a constant weight, Tris–HCl (20 mM, pH 7.4) was used to prepare 10, 20, 30, 40, 50 μg/mL of standard solution. Next, 1 mL of the tyrosine standard solution of the above concentration was taken and 5 mL of 4 mol/L Na_2_CO_3_ solution and 1 mL of the three‐fold diluted Folin Reagent were added. The mixture was heated in a water bath at 40°C for 20 min, the absorbance was measured at 680 nm, and the concentration standard curve of the tyrosine solution was plotted (Figure 2).

**FIGURE 2:**
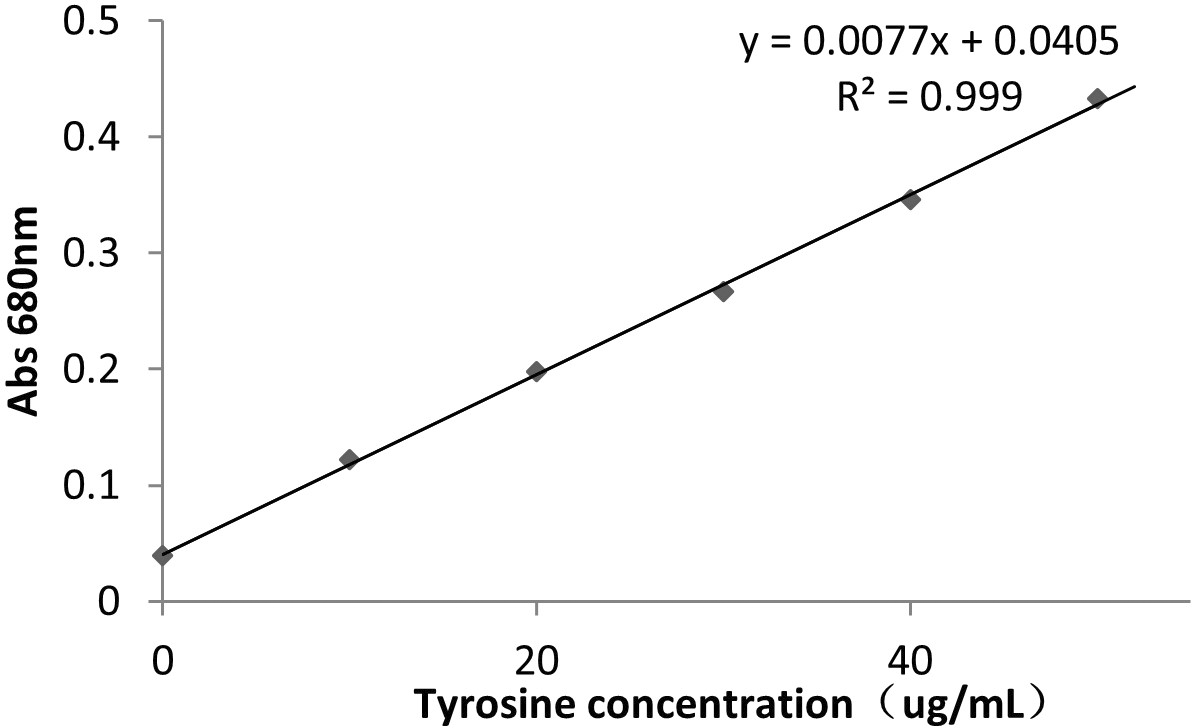
The tyrosine standard curve was determined by the Lowry method.

The lyophilized protease powder in each purification step was prepared into a 2 mg/mL protease solution with 20 mM Tris–HCl (pH 7.4). After preheating at 40°C for 5 min, 1 mL of 2% casein solution was added. After reacting at 40°C for 10 min, 2 mL of 0.4 mol/L trichloroacetic acid solution was added and the mixture was left undisturbed for 5 min to stop the reaction. After filtering with a qualitative filter paper, 1 mL of the filtrate was mixed with 5 mL of 4 mol/L Na_2_CO_3_ solution and (3×) 1 mL of Folin Reagent. After heating in a water bath at 40°C for 20 min, 200 μL of the reacted liquid was added to a 96‐well plate, and the absorbance Ab was measured at 680 nm.

The absorbance of the 2 mg/mL protease freeze‐dried powder solution was measured at 680 nm, and it was designated as A0.

The specific activity unit of protease was defined as follows: Micrograms per minute of casein hydrolyzed to tyrosine by 1 g of enzyme powder at 40°C, pH 7.4, i.e., $A=\frac{\mathrm{Ab}-A0}{K}\times \frac{4}{10}\times \frac{N}{W}$ (pA: Protease specific activity, IU/g; Ab: sample absorbance value at 680 nm; A0: Y (x = 0); 4/10: 4 mL system reaction for 10 min; N: sample dilution factor; W: sample quality in g).

### 2.5 Detection of enzymatic properties

#### 2.5.1 Temperature stability

First, 20 mM Tris–HCl (pH 7.4) was used to prepare plasmin as a 2 mg/mL protease solution. After activating at 40°C for 5 min, 1 mL of 2% casein solution was added, and the mixtures were incubated at 20, 30, and 40°C, respectively. After reacting at 50, 60, 70, and 80°C for 10 min, 2 mL of 0.4 mol/L trichloroacetic acid solution was added immediately and left undisturbed for 5 min to terminate the reaction. After filtering with qualitative filter paper, 1 mL of the filtrate was taken, and the protease activity was determined by following the method described in Section 2.4. The agarose fiber plate method was used to determine the effect of temperature on the protease fibrinolytic activity (following the method described in Section 2.3) by placing the spotted fiber plates at 20, 30, 40, 50, 60, 70, and 80°C, respectively. The samples were incubated for 18 h, and the size of the transparent circle on the plate was recorded.

#### 2.5.2 pH stability

First, 20 mM citric acid‐sodium citrate buffer (pH 3, 4, and 5), 20 mM PBS buffer (pH 6, 7, and 8), and 20 mM Tris–HCl buffer solution (pH 9, 10, and 11) were configured. Then, the above buffer was used to prepare 2 mg/mL protease solution and activate it at the optimum temperature for 5 min, and then, 1 mL of 2% casein solution was added and placed at the optimum pH. After the mixture was allowed to react at an optimum temperature for 10 min, 2 mL of 0.4 mol/L trichloroethane solution was added and left undisturbed for 5 min to terminate the reaction. After filtering with qualitative filter paper, 1 mL of the filtrate was taken, and the protease activity was determined following the method described in Section 2.4.

The agarose fiber plate method was used to determine the effect of different pH values on the protease fibrinolytic activity. The citric acid‐sodium citrate buffer (pH 3, 4, and 5), PBS buffer (pH 6, 7, and 8), and Tris–HCl buffer solution (pH 9, 10, and 11) were configured with 50 mg/mL protease solution, 2% agarose solution, and 1 mg/mL fibrinogen solution. The method described in Section 2.3 was followed to configure fiber plates in different pH environments. The protease samples prepared with the above‐mentioned different pH buffer solutions were activated for 5 min and spotted on fiber plates of different pH, respectively, followed by incubation at the optimal temperature for 18 h. Finally, the size of the transparent circle was recorded.

#### 2.5.3 Stability of the metallic ion

An experiment was performed to assess the content of common metal ions in human blood and design in vitro experiments to determine Clamworm fibrinolytic protease activity.

Using the buffer with the most suitable pH in the result corresponding to Section 2.5.2 as the solvent (Table 1), 148 mM NaCl solution, 5.5 mM KCl solution, 5.5 mM CaCl_2_ solution, 1.0 mM MgCl_2_ solution, and 77 μM FeCl_2_ solution were configured. Additionally, 77 μM FeCl_3_ was used as a control.

**TABLE 1:** Metallic ion concentration in serum (human).

Metallic ion	Concentration in serum
Na^+^	135 ~ 148 mM
K^+^	3.5 ~ 5.5 mM
Ca^2+^	4.5 ~ 5.5 mM
Mg^2+^	0.74 ~ 1.0 mM
Fe^2+^	50 ~ 77 μM

The protease solution (2 mg/mL) was prepared with the metal ion solution mentioned above. After activating at 40°C for 5 min, 1 mL of 2% casein solution was added, incubated at the optimum temperature of the protease, and allowed to react for 10 min. Immediately after the reaction, 2 mL of 0.4 mol/L trichloroacetic acid solution was added, and the mixture was left undisturbed for 5 min to terminate the reaction. After filtering the contents with a qualitative filter paper, 1 mL of filtrate was taken, and the protease activity was determined according to the method described in Section 2.4.

The agarose fiber plate method was used to determine the effect of metal ions on the protease fibrinolytic activity. The above metal ion solution was used to prepare 50 mg/mL protease solution and 2% agarose solution, and they were configured following the method described in Section 2.3. On the fiber plate, the activated protease was spotted separately, followed by incubation at the optimum temperature for 18 h. Finally, the size of the transparent circle on the plate was recorded.

#### 2.5.4 Molecular weight

Following the method described by Ola et al.^24^ denaturing gel electrophoresis on protease was performed to determine the number and molecular weight of its polypeptide chains. The method is as follows:

SDS‐PAGE reagents: 10× denaturing electrode buffer and 5× denaturing loading buffer; SDS‐PAGE electrophoresis gel configuration was as follows:

Sample pretreatment: First, 20 μL of 50 mg/mL sample and 5 μL of denaturation loading buffer were added to the centrifuge tube, and boiled in a water bath at 100°C for 5 min. Then, electrophoresis and color development were performed. The obtained electrophoresis gel was captured with a UVP system to analyze protein bands.

### 2.6 LC–MS–MS analysis of enzymatically cleaved peptides

#### 2.6.1 Experimental materials

The 44.5 and 130 kDa protein bands were separated by Native‐PAGE from Asp60‐D1‐P1 protease in Section 2.2.

#### 2.6.2 Experimental reagents

Trypsin was purchased from Promega. Guanidine isothiocyanate, indoleacetic acid, and pancreatic coagulase were purchased from Sigma. Urea, Tris‐base, and trimethylolamine methane were purchased from Bio‐Rad. Zeba centrifugal desalting column was purchased from Pierce. Protein endonuclease Glu‐c and peptide chain endonuclease Lys‐c were purchased from Wako.

#### 2.6.3 Instruments and equipment

Liquid chromatography system (Easy‐nLC 1000 model, Thermo Fisher, USA); Q Exactive mass spectrometer (Thermo Fisher, USA).

#### 2.6.4 Experimental methods

##### Enzymatic digestion of samples

The 44.5 and 130 kDa electrophoretic bands obtained by Native‐PAGE separation of Asp60‐D1‐P1 were recovered, and the proteases of the two sets of bands were hydrolyzed by adding trypsin at a mass ratio of 1:50 (m/m) for 20 h (constant temperature at 37°C) after being treated with a reductant and an alkylating agent. A Zeba centrifugal desalting column was used to desalt the sample enzymatic product. The desalted enzymatic product was lyophilized, and the lyophilized powder was redissolved in 0.1% formic acid solution, which formed the enzymatic peptide sample. The enzymatic samples of 44.5 and 130 kDa protein bands were designated as Mr_44.45 and Mr_130, respectively, and were stored at −20°C for spare use.

##### QE analysis

The enzymatic samples obtained in Section 2.5 were separated on an Easy‐nLC 1000 liquid chromatography system using a Nano Trap column (0.1 mm × 20 mm). Equilibrium solution: 0.1% formic acid in water and elution solution: 84% acetonitrile in water containing 0.1% formic acid. Next, 95% of the equilibrium solution rinsed the column until the baseline was stabilized, and then, the samples of Mr_44.45 and Mr_130 were uploaded to the column by an autosampler, respectively, and liquid B was eluted at a gradient of 1 H. The samples were separated by LC on an Easy‐nLC 1000 liquid chromatography system using a Nano Trap column (0.1 mm × 20 mm). The peptide fragments separated by LC were burst into misty charged droplets through the electrospray interface and fed as ions to the Q Exactive mass spectrometer for MS/MS–MS analysis.

##### Data acquisition

To evaluate the mass‐to‐charge ratios of peptides/peptide fragments of Mr_44.45 and Mr_130 samples, 20 fragmentation profiles were collected after each full scan (MS2 scan).

##### Data analysis

The LC–MS/MS–MS results (raw file) of Mr_44.45 and Mr_130 were used to search the uniprot_Perinereis_210_20190813.fasta database with the Mascot 2.2 software. The samples were identified by analyzing their proteomic matching results.

## 3 RESULTS AND DISCUSSION

### 3.1 Enzymatic activity of crude protein

In this experiment, the saturation of ammonium sulfate in the crude silkworm protein extract was adjusted to 40%, 60%, and 80% by the sulfate salting‐out method, and the crude silkworm protease concentrated in the three salting‐out sections was recorded as Asp40, Asp60, and Asp80, to determine their protease activity (Table 2) and fibrinolytic activity (Table 3).

**TABLE 2:** The crude protease activity of *Perinereis aibuhitensis* was determined by ammonium sulfate precipitation.

(NH4)_2_SO_4_ salting out sample (2 mg/mL)	Protein content (μg/mg)	Crude extract protease activity (×10^−3^ U/mg)	Crude enzyme protease specific activity (IU/mg)
0%–40% (Asp40)	44.76	363.64	8.12
40%–60% (Asp60)	41.76	467.53	11.19
60%–80% (Asp80)	50.27	597.40	10.85

**TABLE 3:** The crude protease fibrinolytic activity of *Perinereis aibuhitensis* was determined by ammonium sulfate precipitation (Ultraviolet Assay).

(NH4)_2_SO_4_ salting out sample (50 mg/mL)	Protein content (μg/mg)	Crude extract protease activity (U/mg)	Crude enzyme protease‐specific activity (U/mg)
0%–40% (Asp40)	44.76	1.69	37.76
40%–60% (Asp60)	41.76	3.65	87.40
60%–80% (Asp80)	50.27	1.89	37.59

After ammonium sulfate salting out, dialysis, and lyophilization of the crude protease solution, the protein content of Asp40, Asp60, and Asp80 samples were 44.76 μg/mg, 41.76 μg/mg, and 50.27 μg/mg, respectively, and the concentration was 50 mg/mL. The casein hydrolysis activity and fibrin hydrolysis activity of the three groups of samples were quantified. The results showed that 40%–60% (NH_4_)_2_SO_4_ salted‐out protein (Asp60) and 60–80% salted‐out protein (Asp80) had high protease hydrolysis activity, respectively, 11.19 and 10.85 IU/mg (Table 2). The protease in the Asp60 group had the highest fibrinolytic activity (87.4 U/mg), which was 1.31 and 1.32 times that of the Asp40 and Asp80 proteases, respectively (Table 2). The three groups of samples of 10 μL of 50 mg/mL were spotted on the fibrin plate, respectively, and the 18 h culture results showed that the transparent circles of the Asp40 and Asp80 protease groups were of the same size, and both were significantly smaller than the transparent circles of the Asp60 group (Figure 3). The results were consistent with the UV quantitative results of the three groups of protease fibrinolytic activity. Therefore, the Asp60 group of proteases was selected for subsequent purification steps.

**FIGURE 3:**
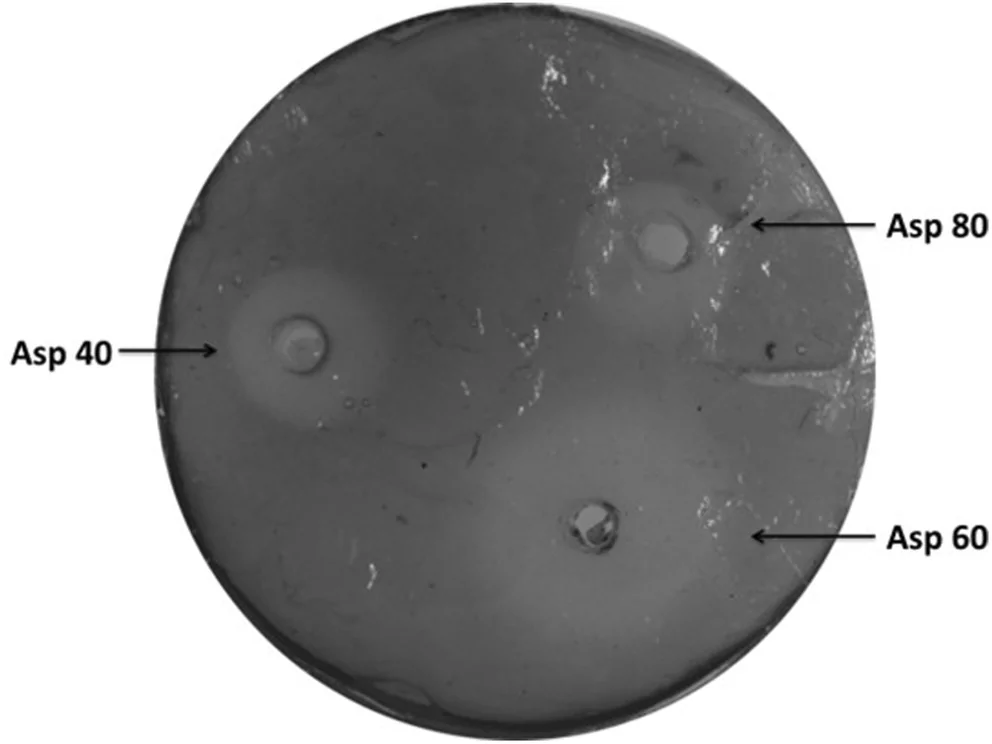
The crude protease fibrinolytic activity of *Perinereis aibuhitensis* was determined by ammonium sulfate precipitation (Fibrin Agarose Plate Assay).

### 3.2 Primary chromatography enzyme activity analysis

The protease Asp60, which had the highest fibrinolytic activity and enzyme activity after salting out, was used for fractional chromatography. After Asp60 passed through the Sephadex G‐100 gel column, two prominent protein peaks, Asp60‐G1 and Asp‐G2, appeared in the elution profile (Figures 4, 5, 6). After passing through the DEAE‐Sepharose FF gel column, two prominent protein peaks were found. Two prominent protein peaks, Asp60‐D1 and Asp60‐D2, were detected; after the samples were separated using the Phenyl‐Sepharose FF gel column, two unclear protein peaks, Asp60‐P1 and Asp60‐P2, were detected. After lyophilizing the separated protein peak samples, the fibrinolytic activity and protease activity were determined (Table 2), and the three proteins with higher fibrinolytic activity were selected under the three chromatographic methods. Peaks: Asp60‐G1, Asp60‐D1, and Asp60‐P2 for the second step of chromatography.

**FIGURE 4:**
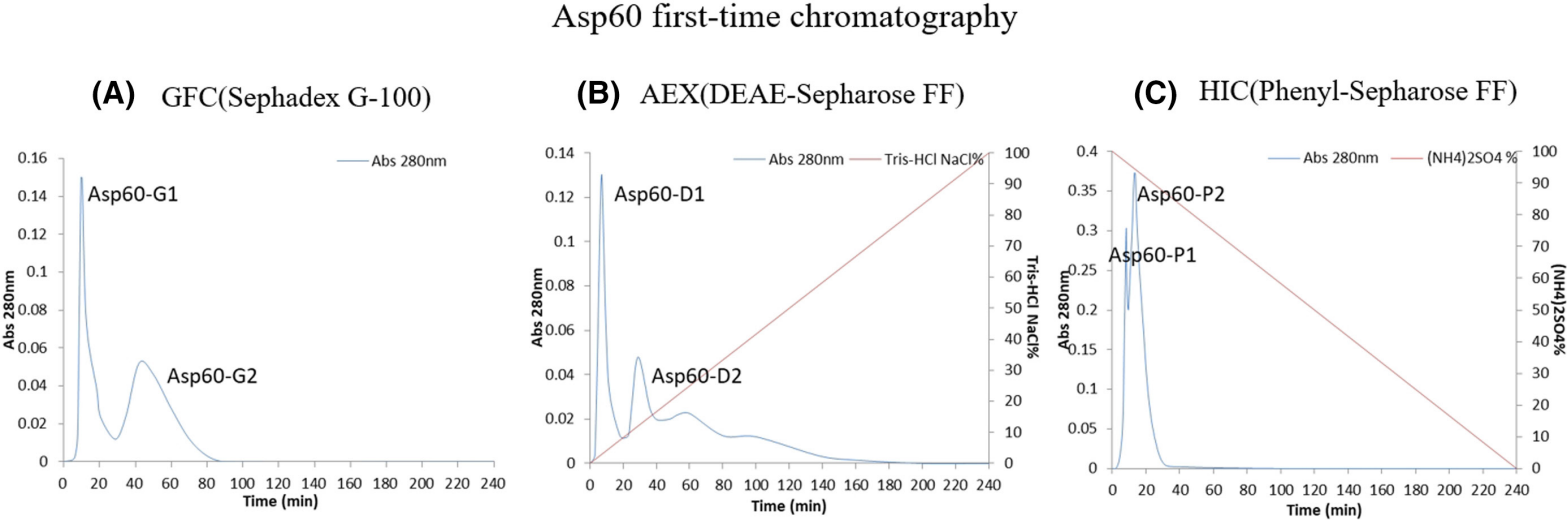
Chromatography of 40–60% ammonium sulfate salting protease (Asp60). (A): GFC (Sephadex G‐100); (B): AEX (DEAE‐Sepharose FF); (C): HIC (Phenyl‐Sepharose FF).

Asp60 was separated by first‐level chromatography. The protease enzyme activity of the protein peak freeze‐dried powder is shown in Table 4. The Sephadex G‐100 and DEAE‐Sepharose FF chromatographic columns had better separation effects on Asp60, and the total purified protease activities were 5.48 and 5.36 times compared to that before purification. The specific activity of the Asp60‐D1 protein peak was the highest among all separated peaks (49.79 IU/mg), followed by Asp60‐G1 (31.50 IU/mg). Hydrophobic chromatography was performed as First‐level chromatography, the protein peak of Asp60 on the Phenyl‐sepharose FF column was not clearly separated, and the protease activity of the separated peak was low (4.92 and 11.69 IU/mg, respectively). Thus, hydrophobic chromatography was used as the first‐level chromatography, which had a poor separation effect.

**TABLE 4:** The protease activity of Asp60 was determined by first‐level chromatography.

Purification steps		Protein content (μg/mg)	Crude extract protease activity (×10^−3^ U/mg)	Purified protease specific activity (IU/mg)
Asp60 gel filtration chromatography (2 mg/mL)	Peak1 (Asp60‐G1)	16.49	519.48	31.50
	Peak2 (Asp60‐G2)	7.83	233.77	29.86
	Total protease activity	–	–	61.36
Asp60 anion exchange chromatography (2 mg/mL)	Peak1 (Asp60‐D1)	22.43	1116.88	49.79
	Peak2 (Asp60‐D2)	15.33	155.84	10.17
	Total protease activity	–	–	59.96
Asp60 hydrophobic chromatography (2 mg/mL)	Peak1 (Asp60‐P1)	42.23	207.79	4.92
	Peak2 (Asp60‐P2)	46.65	545.45	11.69
	Total protease activity	–	–	16.61

The fibrinolytic activity of the Asp60 protein separation peak after first‐level chromatography is shown in Table 5. The total fibrinolytic activity of the protease after purification by the Sephadex G‐100 and DEAE‐Sepharose FF columns was 1.55 and 2.49, respectively, before purification. The fibrinolytic activity of the Asp60‐D1 protein peak was the highest (167.31 U/mg), which was 1.91 times that of Asp60. The fibrinolytic activity of Asp60‐G1 was equivalent to Asp60. On the Phenyl‐Sepharose FF column, the fibrinolytic activity of the protease separation peaks Asp60‐P1 and Asp60‐P2 decreased by 93% and 70%, respectively. This occurred probably due to the lower salinity of Asp60 protein after dialysis. As hydrophobic chromatography is suitable for the downstream purification of undialyzed salted‐out protein or ion‐exchange chromatography, the fibrinolytic component of Asp60 with low salinity may be bound to the gel filler and not effectively washed off.

**TABLE 5:** The fibrinolytic activity of Asp60 was determined by first‐level chromatography.

Purification steps		Protein content (μg/mg)	Lyophilized powder fibrinolytic activity (U/mg)	Purified protease fibrinolytic activity (U/mg)
Asp60 gel filtration chromatography (50 mg/mL)	Peak1 (Asp60‐G1)	16.49	1.35	81.87
	Peak2 (Asp60‐G2)	7.83	0.42	53.50
	Total fibrinolytic activity	–	–	135.37
Asp60 anion exchange chromatography (50 mg/mL)	Peak1 (Asp60‐D1)	22.43	3.75	167.31
	Peak2 (Asp60‐D2)	15.33	0.78	50.87
	Total fibrinolytic activity	–	–	218.18
Asp60 hydrophobic chromatography (50 mg/mL)	Peak1 (Asp60‐P1)	42.23	0.23	5.45
	Peak2 (Asp60‐P2)	46.65	0.95	20.36
	Total fibrinolytic activity	–	–	25.81

Among all the protein peaks separated by the first‐level chromatography of Asp60, Asp60‐D1 had the highest protease fibrinolytic activity, followed by Asp60‐G1, and the protein peaks Asp60‐G2/D2 had the highest protease fibrinolytic activity (Figure 5). The solubility was relatively weak, and the protein peak obtained by hydrophobic chromatography Phenyl‐Sepharose FF chromatographic column had almost no fibrinolytic activity. These results were similar to those presented in Table 5.

**FIGURE 5:**
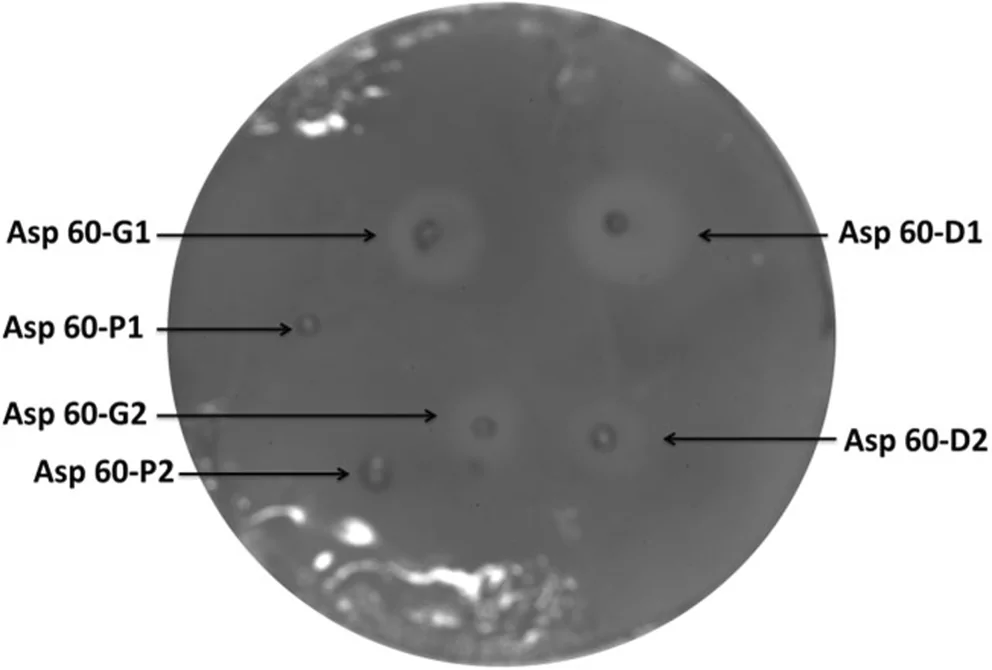
The fibrinolytic activity of Asp60 protease (*Perinereis aibuhitensis*) was determined by first‐level chromatography (Fibrin Agarose Plate Assay).

### 3.3 Secondary chromatography enzyme activity analysis

The chromatographic results of the Asp60‐G1 group protease are shown in Figure 6. The results of DEAE‐Sepharose FF column chromatography showed that Asp60‐G1 exhibited two distinct protein elution peaks, i.e., Asp60‐G1‐D1 and Asp60‐G1‐D1. After Phenyl‐Sepharose FF column chromatography, Asp60‐G1 displayed three protein elution peaks, i.e., Asp60‐G1‐P1, Asp60‐G1‐P2, and Asp60‐G1‐P3. Of the latter two protein elution peaks, the separation of off‐peak was not prominent.

**FIGURE 6:**
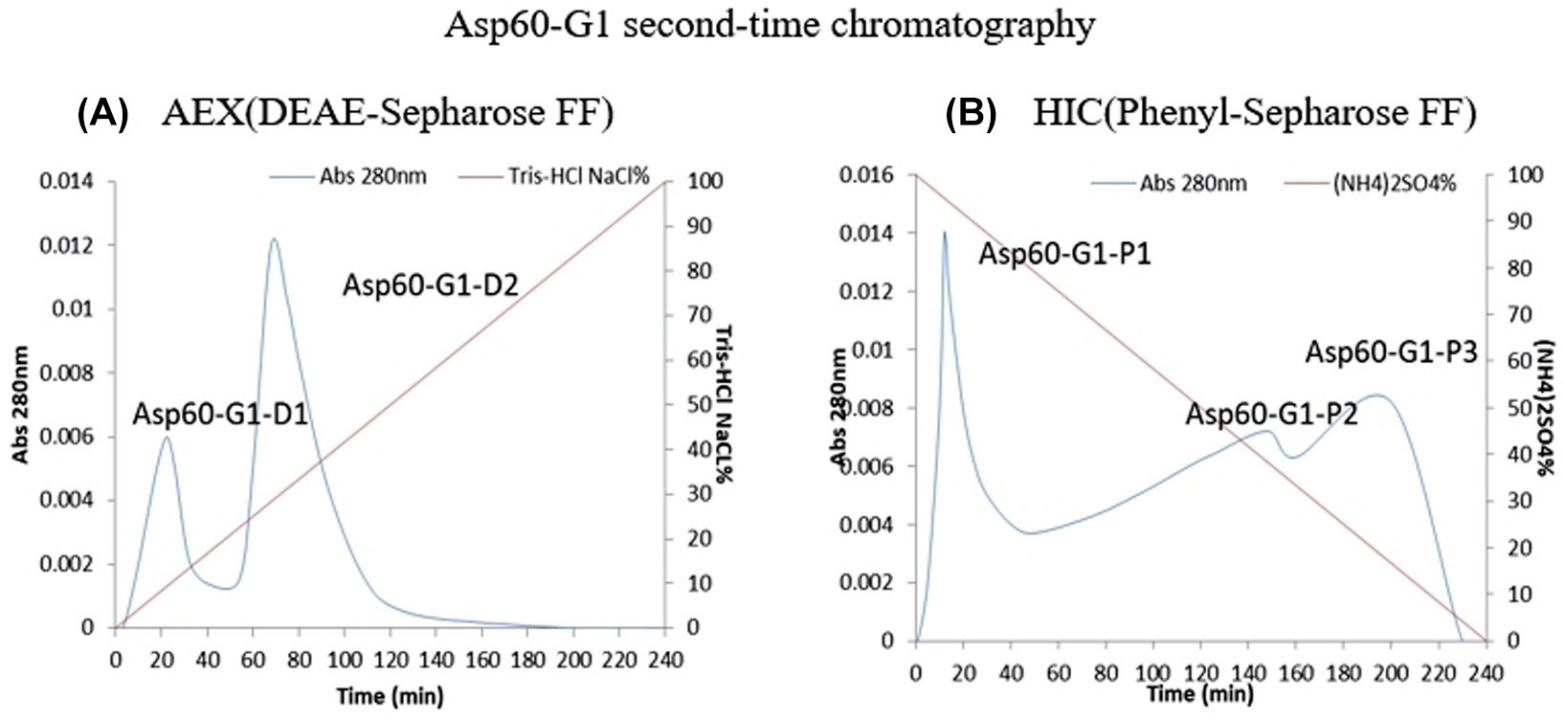
Chromatography of ASP‐G1. (A): AEX (DEAE‐Sepharose FF); (B): HIC (Phenyl‐Sepharose FF).

The chromatographic results of the Asp60‐D1 group protease are depicted in Figure 7. Following Sephadex G‐100 column chromatography, Asp60‐D1 had two close protein elution peaks, i.e., Asp60‐D1‐G1 and Asp60‐D1‐G2, and the retention time of the first group was shorter; thus, the content of this component in Asp‐D1 was less. After Phenyl‐Sepharose FF column chromatography, Asp60‐D1 was eluted out of two distinct protein monomers. The peaks detected were Asp60‐D1‐P1 and Asp60‐D1‐P2, respectively.

**FIGURE 7:**
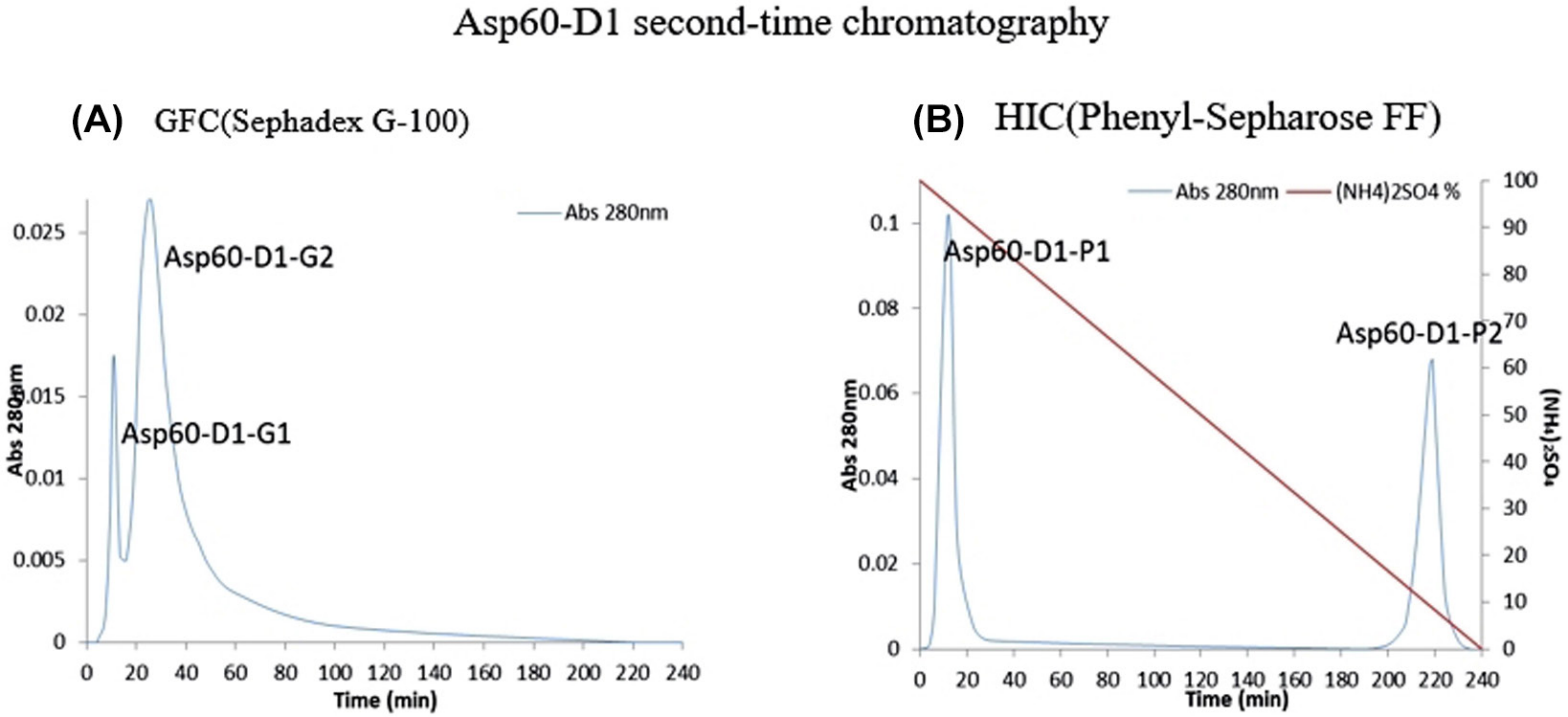
Chromatography of Asp‐D1. (A): GFC (Sephadex G‐100); (B): HIC (Phenyl‐Sepharose FF).

The chromatographic results of the Asp60‐P2 group of proteases are shown in Figure 8. After Asp60‐P2 was passed through the Sephadex G‐100 and DEAE‐Sepharose FF columns, the elution patterns showed single peaks, respectively, Asp‐P2‐G1 and Asp‐P2‐D1, which indicated that the protease component retained by the 40%–60% salted‐out protein of the bidentate silkworm after passing through the hydrophobic gel chromatography column was single.

**FIGURE 8:**
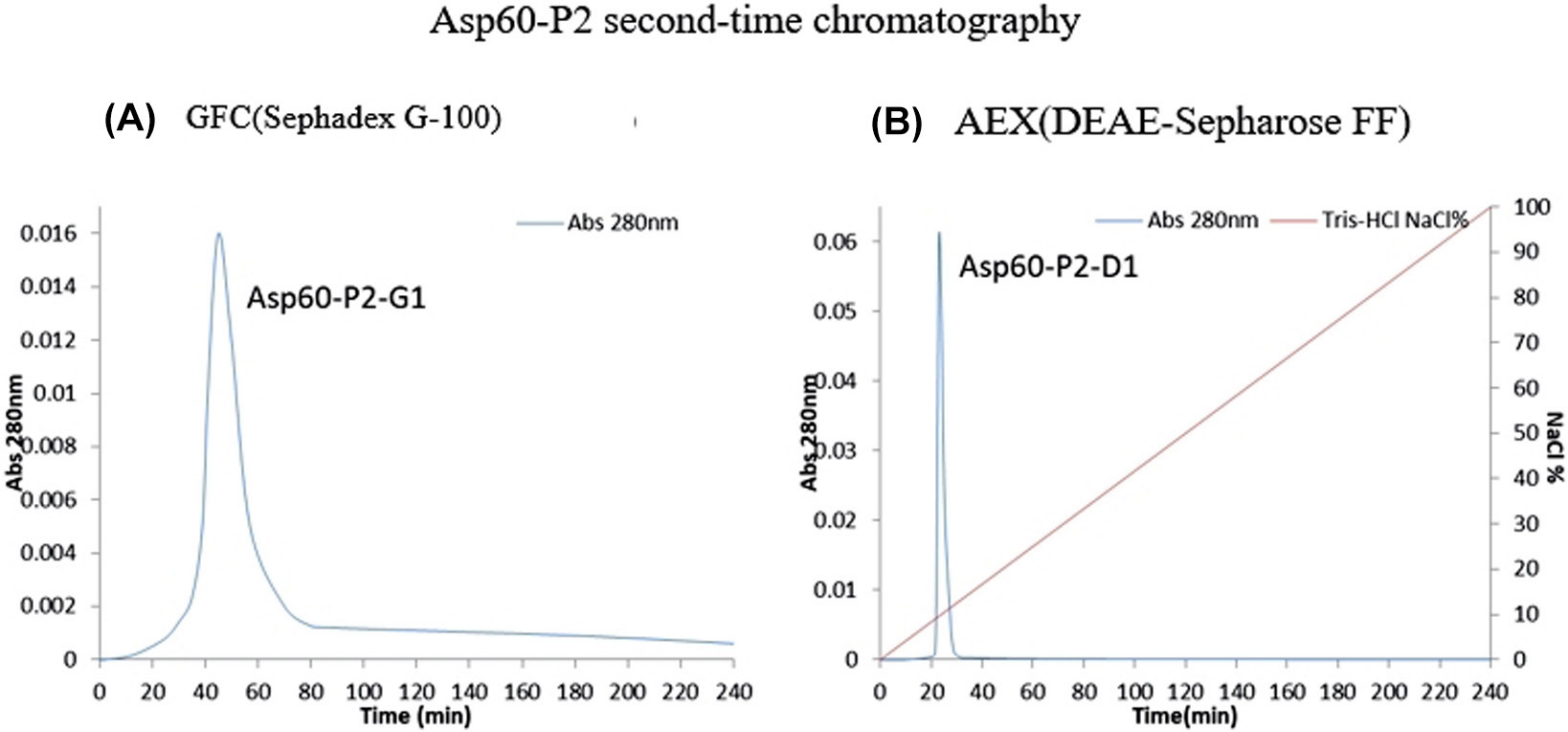
Chromatography of Asp‐P2. (A): GFC (Sephadex G‐100); (B): AEX (DEAE‐Sepharose FF).

After primary chromatography, the protein peaks Asp60‐G1, Asp60‐D1, and Asp60‐P2 with higher activity under the three chromatography methods were selected for the second‐level chromatography. The protease activity of the separated peaks is shown in Table 6. After Asp60‐G1 underwent anion exchange chromatography and hydrophobic chromatography, the total protease activity was 2.40 times and 4.12 times that recorded before purification, respectively. The second separation peak (Asp‐G1) occurred after purification by the Phenyl‐Sepharose FF column‐P2. The specific activity of the protease was higher (77.92 IU/mg), and the third peak Asp‐G1‐P3 showed almost no casein hydrolysis activity.

**TABLE 6:** The protease activity of Asp60 by second‐time chromatography.

Purification steps		Protein content (μg/mg)	Crude extract protease activity (×10^−3^ U/mg)	Purified protease specific activity (IU/mg)
Asp60‐G1 anion exchange chromatography (2 mg/mL)	Peak1 (Asp60‐G1‐D1)	0.87	22.48	25.83
	Peak2 (Asp60‐G1‐D2)	1.91	95.19	49.84
	Total protease activity	–	–	75.67
Asp60‐G1 hydrophobic chromatography (2 mg/mL)	Peak1 (Asp60‐G1‐P1)	0.23	11.95	51.96
	Peak2 (Asp60‐G1‐P2)	0.52	40.52	77.92
	Peak3 (Asp60‐G1‐P3)	0.18	0.00	0.00
	Total protease activity	–	–	129.88
Asp60‐D1 gel filtration chromatography (2 mg/mL)	Peak1 (Asp60‐D1‐G1)	4.51	77.92	17.28
	Peak2 (Asp60‐D1‐G2)	7.34	662.34	90.24
	Total protease activity	–	–	107.52
Asp60‐D1 hydrophobic chromatography (2 mg/mL)	Peak1 (Asp60‐D1‐P1)	14.52	3367.76	231.94
	Peak2 (Asp60‐D1‐P2)	13.98	12.99	0.93
	Total protease activity	–	–	232.87
Asp60‐P2 gel filtration chromatography (2 mg/mL)	Peak1 (Asp60‐P2‐G1)	0.87	16.11	18.52
	Total protease activity	–	–	18.52
Asp60‐P2 anion exchange chromatography (2 mg/mL)	Peak1 (Asp60‐P2‐D1)	0.23	5.39	23.43
	Total protease activity	–	–	23.43

Asp60‐D1 separated two protein peaks by Seprodex G‐100 (Figure 7). As the retention time was close to the overlap between the peaks, the first separated peak Asp60‐D1‐G1 had a smaller peak area and lower casein hydrolysis activity (17.28 IU/mg), while the second separation peak Asp‐D1‐G2 had a larger peak area and higher protease specific activity (90.24 IU/mg), which was 1.81 times the protease activity before purification; Asp60‐D1 Phenyl‐Sepharose FF purified two distinct protein peaks. The specific activity of Asp‐D1‐P1 was high (231.94 IU/mg), and the purification factor was as high as 20.73. The second peak, Asp‐D1‐P2, had almost no casein hydrolytic activity.

After Asp60‐P1 was further analyzed by chromatography, the spectrum showed a single peak (Figure 8), which indicated that the protein component of Asp‐P1 was single, and the results of protease activity showed that this component had almost no casein hydrolysis activity and was a non‐enzymatic protein.

The results of the fibrinolytic activity of Asp60 second‐stage chromatographic protease are shown in Table 7. Asp60‐G1 was analyzed by anion exchange chromatography, and the fibrinolytic activity of the second protein peak was 136.13 U/mg, which was higher than that of Asp60‐G1. Vitality was increased by 66%. The results of hydrophobic chromatography showed that the total fibrinolytic activity of the separated peak of Asp60‐G1 was lost, and the loss rate was about 29.53%.

**TABLE 7:** The fibrinolytic activity of Asp60 was determined by second‐time chromatography.

Purification steps		Protein content (μg/mg)	Lyophilized powder fibrinolytic activity (U/mg)	Purified protease fibrinolytic activity (U/mg)
Asp60‐G1 anion exchange chromatography (50 mg/mL)	Peak1 (Asp60‐G1‐D1)	0.87	0.02	–
	Peak2 (Asp60‐G1‐D2)	1.91	0.26	136.13
	Total fibrinolytic activity	–	–	136.13
Asp60‐G1 hydrophobic chromatography (50 mg/mL)	Peak1 (Asp60‐G1‐P1)	0.23	0.01	–
	Peak2 (Asp60‐G1‐P2)	0.52	0.03	57.69
	Peak2 (Asp60‐G1‐P3)	0.18	0.02	–
	Total fibrinolytic activity	–	–	57.69
Asp60‐D1 gel filtration chromatography (50 mg/mL)	Peak1 (Asp60‐D1‐G1)	4.51	0.20	44.35
	Peak2 (Asp60‐D1‐G2)	7.34	0.33	44.96
	Total fibrinolytic activity	–	–	89.31
Asp60‐D1 hydrophobic chromatography (50 mg/mL)	Peak1 (Asp60‐D1‐P1)	14.52	2.93	201.79
	Peak2 (Asp60‐D1‐P2)	13.98	0.36	22.17
	Total fibrinolytic activity	–	–	223.96
Asp60‐P2 gel filtration chromatography (50 mg/mL)	Peak1 (Asp60‐P2‐G1)	0.87	0.01	–
	Total fibrinolytic activity	–	–	–
Asp60‐P2 anion exchange chromatography (50 mg/mL)	Peak1 (Asp60‐P2‐D1)	0.23	0.02	–
	Total fibrinolytic activity	–	–	–

Asp60‐D1 was purified by Phenyl‐Sepharose FF. The fibrinolytic activity of the first protein separation peak Asp‐D1‐P1, was the highest among all protein peaks of Asp60 secondary chromatography, which was 201.79 U/mg. The fibrinolytic activity of Asp60‐D1 and Asp60‐D1 were 1.31 times and 0.21 times higher than that of Asp60, respectively. The two protein peaks (Asp60‐D1‐G1 and Asp60‐D1‐G2) were obtained by Sephadex G‐100 column separation only before purification (27% of fibrinolytic activity).

The Asp‐G1‐D1, Asp60‐G1‐P1/P3, and Asp60‐P2‐G1/D1 five groups of chromatographically separated peaks with lower protease content in lyophilized powder, and the degradation products of hydrolyzed fibrin had light absorbance at 273 nm. Therefore, the fibrinolytic enzyme activity calculated based on the fibrinolytic activity standard curve was relatively high, and the qualitative results of the fiber plate showed that these five groups of proteases had almost no fibrinolytic activity (Figure 9). This disparity may be caused by the method error of the ultraviolet method, and thus, we did not discuss the quantitative aspects of the fibrinolytic activity of these five groups of proteases.

**FIGURE 9:**
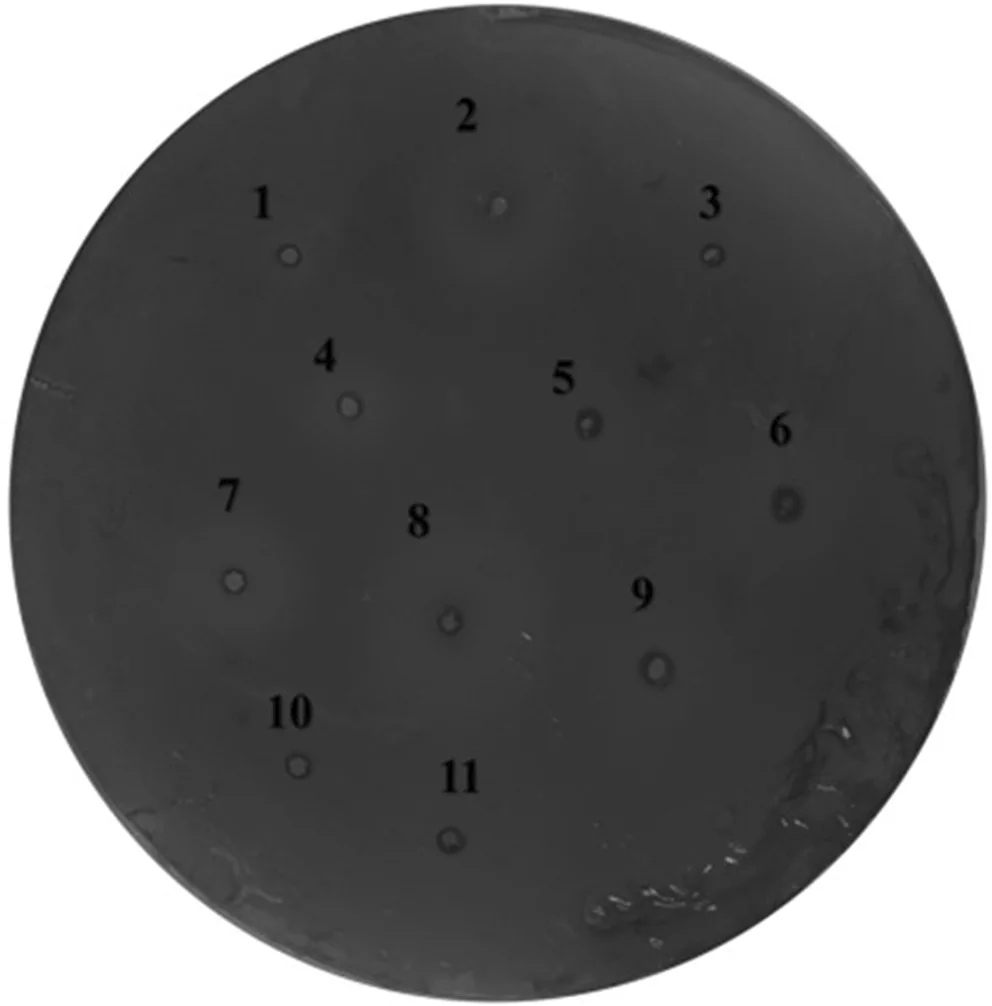
The fibrinolytic activity of Asp60 protease (*Perinereis aibuhitensis*) by second‐time chromatography (Fibrin Agarose Plate Assay, 1:Asp60‐G1‐D1; 2: Asp60‐G2‐D2; 3: Asp60‐G1‐P1; 4: Asp60‐G1‐P2; 5: Asp60‐G1‐P3; 6: Asp60‐D1‐G1; 7: Asp60‐D1‐G1; 8: Asp60‐D1‐P1; 9: Asp60‐D1‐P2; 10: Asp60‐P1‐G1; 11: Asp60‐P1‐D1, Loading quantity: loading 10 μL of 50 mg/mL sample in each hole).

The qualitative results of the fibrinolytic activity of the protein peak obtained by second‐level chromatography at Asp60 (fiber plate method) are shown in Figure 9. The fibrinolytic activity of Asp60‐D1‐P1 and Asp60‐G1‐D2 in the purified protein peak was relatively strong. Asp60‐D1‐G1, Asp60‐D1‐P1, and Asp60‐D1‐P2 exhibited the same fibrinolytic activity, and they were significantly higher than the other purified protein peaks. The protease obtained through further chromatography of Asp‐P1 had no fibrinolytic activity. To summarize, the qualitative results of the protease fibrinolytic activity in the second‐stage chromatography of Asp60 (Figure 9) matched the quantitative results (Table 7). Asp60‐D1‐P1 exhibited fibrinolytic activity in the protein peaks in the second‐stage chromatography and showed the highest enzyme activity.

### 3.4 Three‐stage chromatography enzyme activity analysis

The purified protein peaks with high secondary chromatographic activity were collected and lyophilized, and the protein content, protease activity (Table 8), and fibrinolytic activity (Table 9) of each lyophilized powder were determined.

**TABLE 8:** The protease activity of Asp60 was determined by third‐time chromatography.

Purification steps		Protein content (μg/mg)	Crude extract protease activity (×10^−3^ U/mg)	Purified protease specific activity (IU/mg)
Asp60‐D1‐P1 gel filtration chromatography (2 mg/mL)	Peak1 (Asp60‐D1‐P1‐G1)	0.63	52.59	83.48
	Total protease activity	–	–	83.48

**TABLE 9:** The fibrinolytic activity of Asp60 was determined by second‐time chromatography.

Purification steps		Protein content (μg/mg)	Lyophilized powder fibrinolytic activity (U/mg)	Purified protease fibrinolytic activity (U/mg)
Asp60‐D1‐P1 Gel filtration chromatography (50 mg/mL)	Peak1 (Asp60‐D1‐P1‐G1)	0.63	0.04	63.49
	Total fibrinolytic activity	–	–	63.49

Asp60‐D1‐P1, with the highest fibrillation activity, was selected among the second‐stage chromatographic proteases for gel filtration chromatography, and a protein peak enzyme was separated and purified (Figure 10), which indicated the protein component of Asp60‐D1‐P1 has a single protein fraction. The protease activity of the third stage purification peak is about 35.99% of the pre‐purification level, with a high loss rate, which indicated that the protease with higher purity and activity could be separated by anion exchange chromatography and hydrophobic chromatography. Further chromatography purification led to the loss of yield and activity of the enzyme.

**FIGURE 10:**
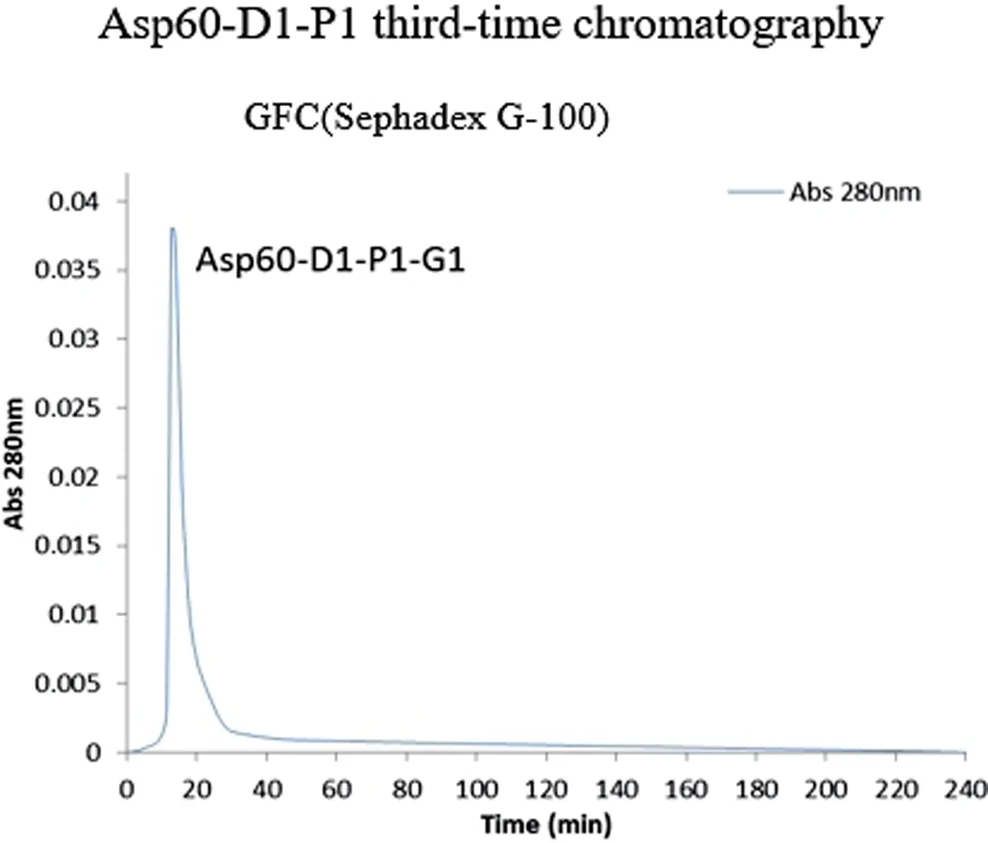
Chromatography of Asp‐D1‐P1.

The third step of chromatography (gel filtration chromatography) was performed on Asp60‐D1‐P1, which had the highest fibrinolytic activity in the protein peak of Asp60 second‐level chromatography. The results showed that Asp60‐D1‐P1 was separated into a single protein by the Sephadex column. The qualitative results of the fibrinolytic activity (Figure 11) of the Asp60‐D1‐P1‐G1 peak (Figure 10) showed that the enzyme had fibrinolytic activity; the quantitative results (Table 9) showed that the third‐level chromatography caused a loss of about 68.53% of the fibrinolytic activity of the enzyme. The results presented in Table 8 also showed that this step of purification caused a loss of 64.01% of the protease activity. To summarize, the fibrinolytic active protease Asp60‐D1‐P1 with a single component and higher activity were obtained by Asp60 through anion exchange chromatography and hydrophobic chromatography. The third‐stage chromatography significantly decreased the enzyme activity and was not suitable for adding bidentate to purify the fibrinolytic active protease of the larvae (Figure 12).

**FIGURE 11:**
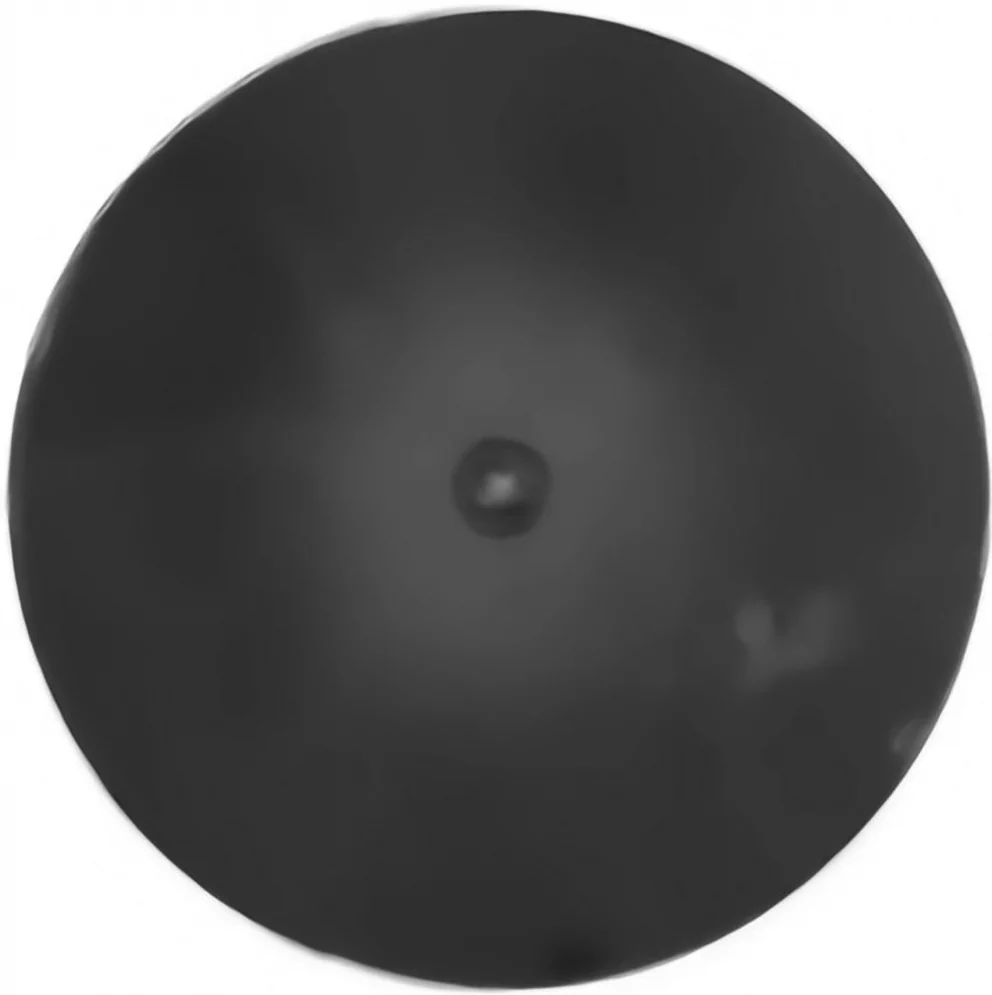
Third‐time chromatography (Fibrin Agarose Plate Assay).

### 3.5 Temperature stability analysis

This temperature gradient of 20–80°C was set at 10°C. Then, the effect of temperature on the activity of Asp60‐D1‐P1 was determined by the tyrosine hydrolysis method and fiber plate method, and the temperature stability of the enzyme was assessed (Figure 12). After activation of Asp60‐D1‐P1, the hydrolysis activity of casein at 20–80°C first increased and then decreased. When the reaction temperature was 40°C, its protease activity was the highest. When the temperature exceeded 60°C, the enzyme activity dropped rapidly. When the reaction temperature was 80°C, the enzyme activity was lost. To summarize, Asp60‐D1‐P1 could maintain good protease activity at 20–60°C, and its optimum activity was 40°C. The transparent circle results corresponding to the fiber plate method in Figure 13 also showed that Asp60‐D1‐P1 had fibrinolytic activity at 30–60°C, and the fibrinolytic activity was the strongest at 40°C. When the temperature exceeded 70°C, the enzyme had no fibrinolytic activity, which matched the result of the stability of the enzyme protease activity.

**FIGURE 12:**
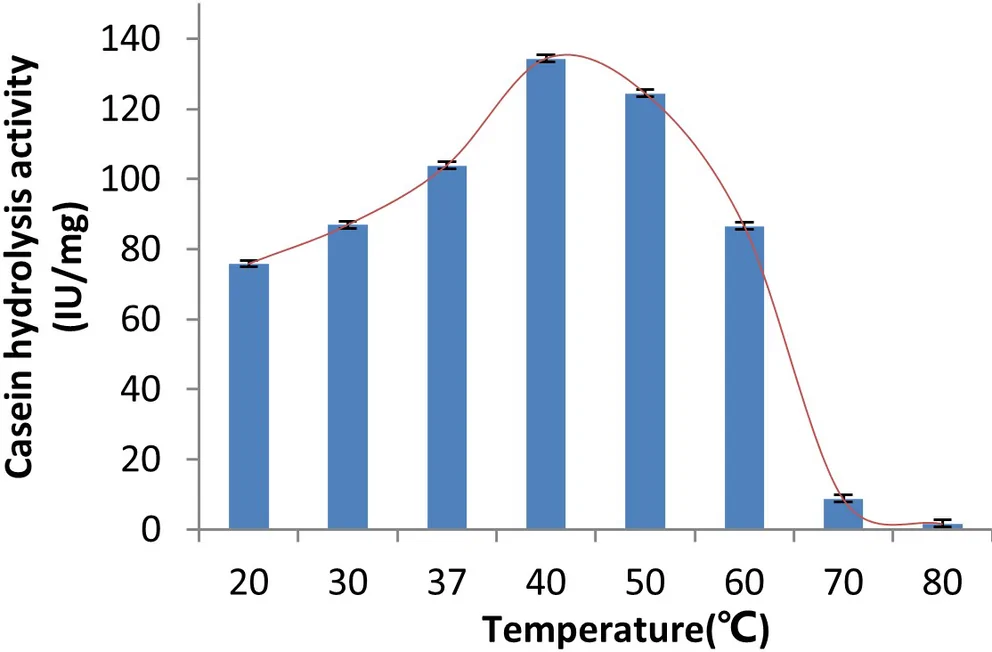
The protease activity of Asp60‐D1‐P1 at different temperatures (20–80°C).

**FIGURE 13:**
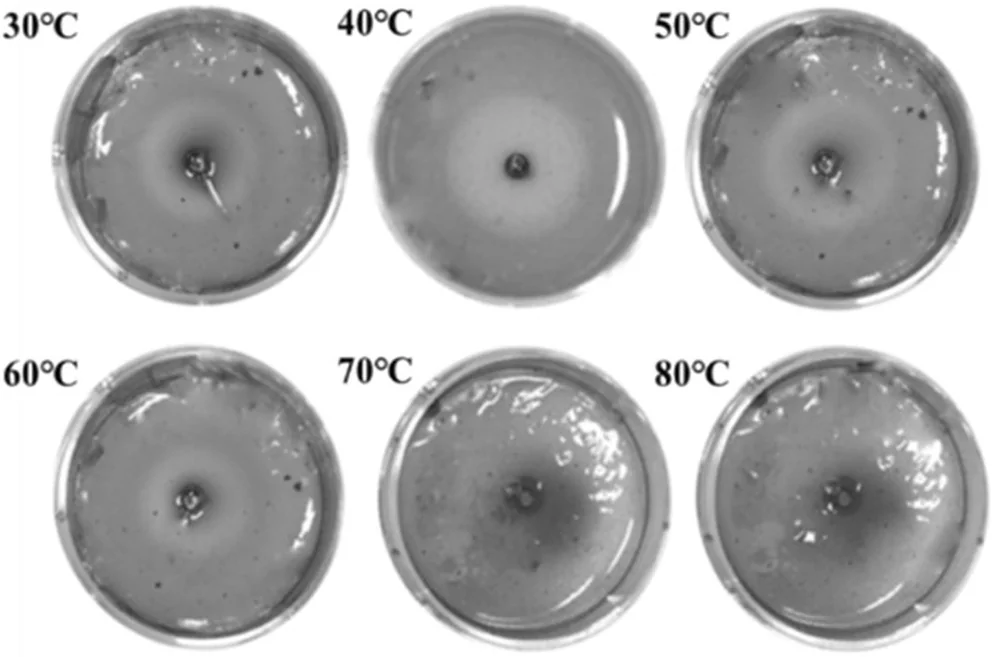
The fibrinolytic activity of Asp60‐D1‐P1 at different temperatures (20–80°C).

### 3.6 pH stability analysis

In this experiment, different buffer solutions of pH 3.0–11.0 were used to dissolve Asp60‐D1‐P1 to become 2 mg/mL of enzyme solution, and the protease activity was determined by tyrosine hydrolysis method. The results are shown in Figure 14. Asp60‐D1‐P1 showed a double S curve in the protease activity in the pH range of 3.0–11.0, and the first peak of protease activity appeared at pH 8.0. The enzyme activity was lower under acidic conditions, and the enzyme was lost at pH 3.0. As the enzyme activity was relatively high under alkaline conditions, the enzyme was speculated to be alkaline protease. The fibrinolytic activity of Asp60‐D1‐P1 under pH 3.0–11.0 conditions is shown in Figure 15. When the pH was less than 5.0, the enzyme had almost no fibrinolytic activity. When the pH was 3.0, the fiber slab could not molded. When the pH was 6.0–11.0, the enzyme had fibrinolytic activity, and the fibrinolytic activity was stronger under alkaline conditions. This result matched the protease activity result, which indicated that Asp60‐D1‐P1 is an alkaline protease and showed optimum activity at pH 8.0.

**FIGURE 14:**
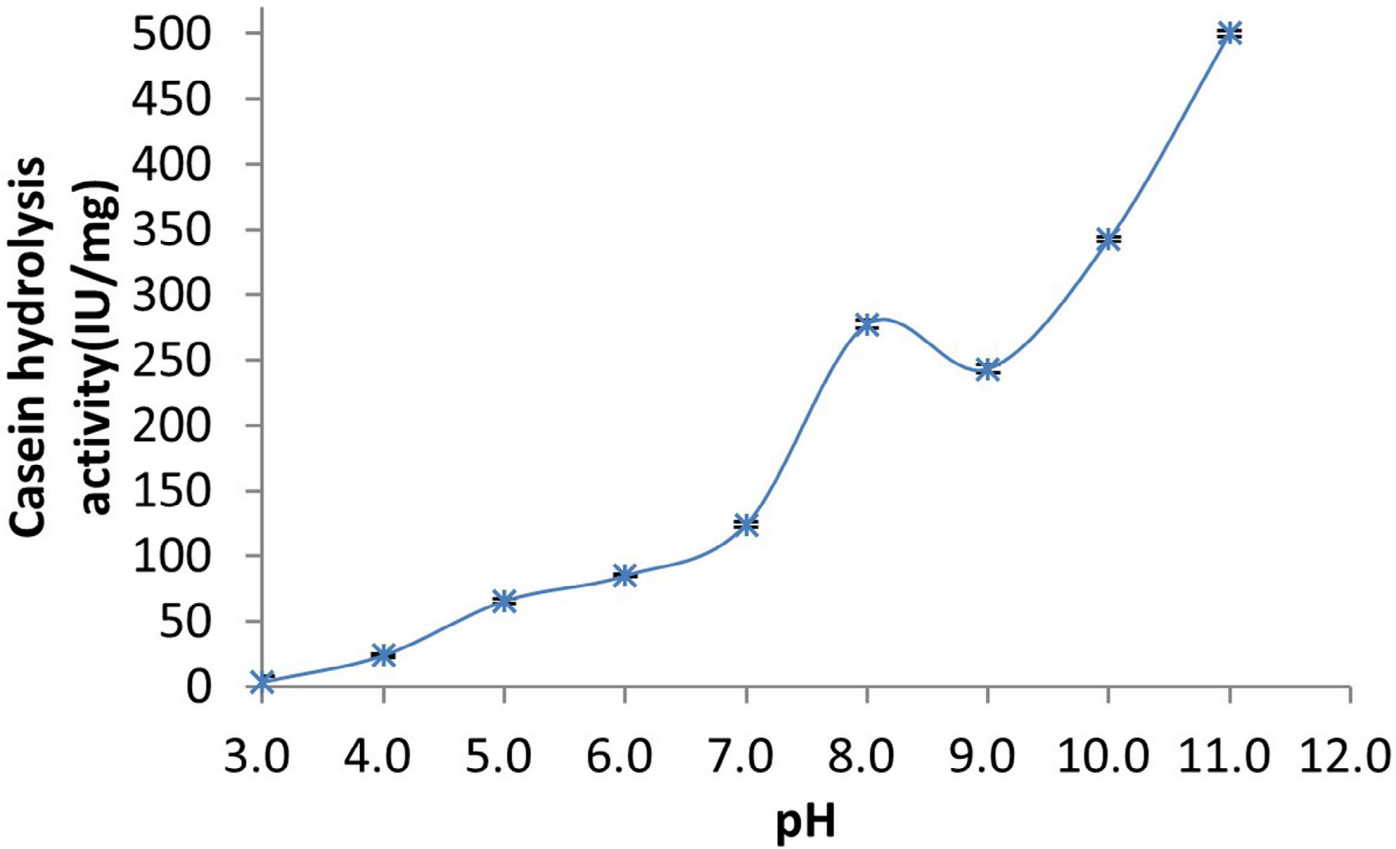
The protease activity of Asp60‐D1‐P1 at different pH (pH 3.0–11.0).

**FIGURE 15:**
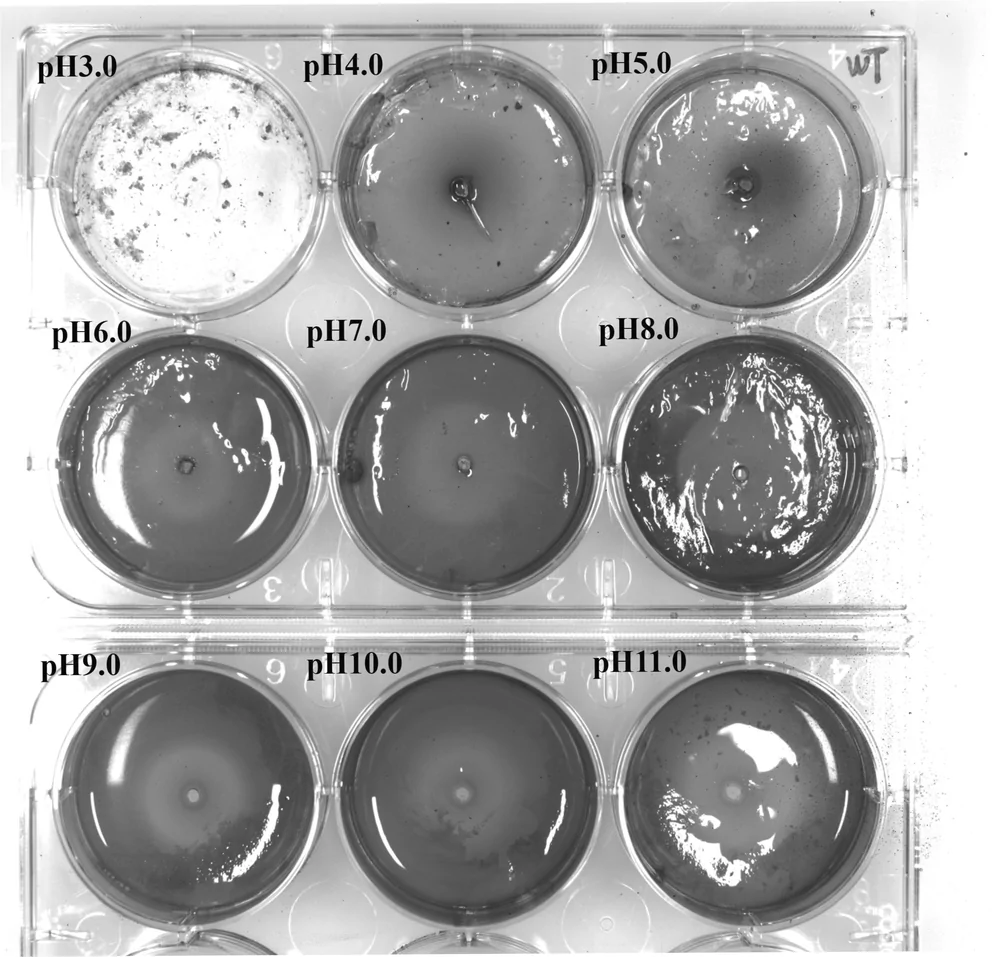
The fibrinolytic activity of Asp60‐D1‐P1 at different pH (pH 3.0–11.0).

### 3.7 Metallic ion stability analysis

In this experiment, we determined the effects of five common metal ions (Na^+^, K^+^, Ca^2+^, Mg^2+^, and Fe^2+^) and Fe^3+^ present in human serum on the activity of Asp60‐D1‐P1. The protease activity of Asp60‐D1‐P1 was equivalent to that found in the control group (NA group) under the environment of 148 mM Na^+^ and 1.0 mM Mg^2+^ (Figure 16). Additionally, 5.5 mM K^+^ and 77 μM Fe^2+^ slightly increased the casein hydrolysis activity of the enzyme. Moreover, 5.5 mM Ca^2+^ slightly inhibited the activity of the enzyme, while the enzyme had almost no protease activity in the 77 μM Fe^3+^ environment. The results of the fiber plate (Figure 17) showed that Asp60‐D1‐P1 was relatively stable in the environment containing Na^+^, K^+^, Ca^2+^, Mg^2+^, Fe^2+^, and Fe^3+^, which significantly inhibited the fibrinolytic activity of the enzyme. This result was similar to that of protease. The vitality results were similar. To summarize, the fibrinolytic activity of Asp60‐D1‐P1 protease in the blood circulation was negligibly affected by metal ions, while Fe^3+^ significantly inhibited the protease and fibrinolytic activity of Asp60‐D1‐P1 and acted as an inhibitor of this enzyme.

**FIGURE 16:**
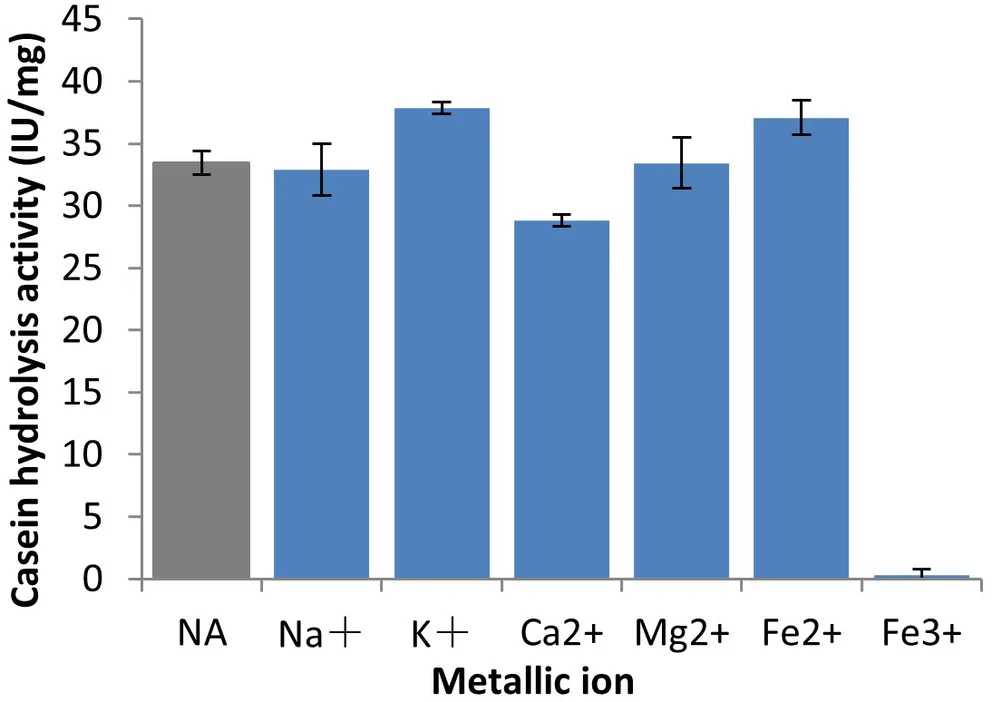
The effect of metal ions on the protease activity of Asp60‐D1‐P1 (NA: Blank group).

**FIGURE 17:**
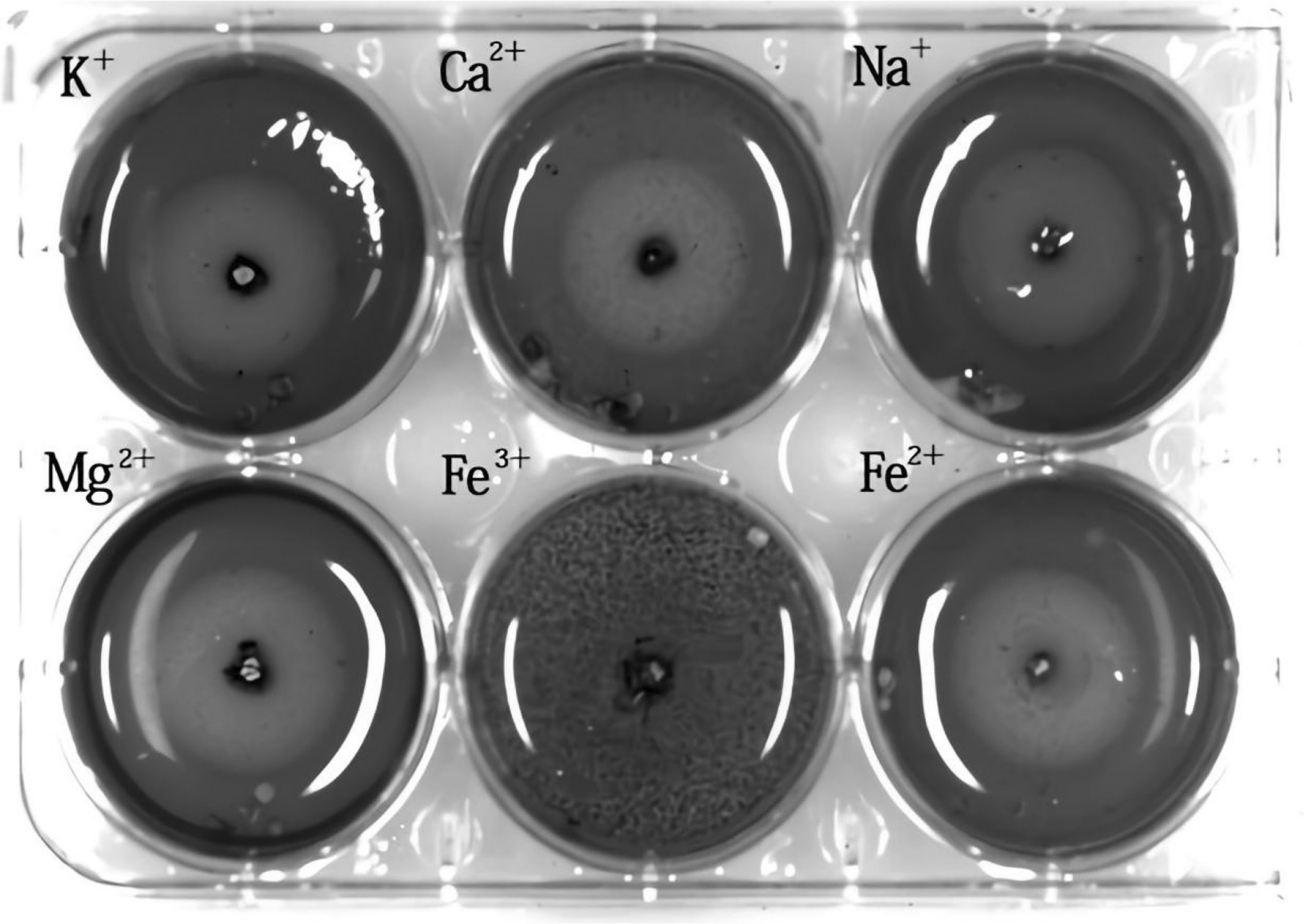
The effect of metal ions on the fibrinolytic activity of Asp60‐D1‐P1.

### 3.8 Molecular weight analysis

Polypropylene gel electrophoresis was performed to estimate the purity, protein molecular weight, protein polypeptide chain composition, and molecular weight of each peptide chain of Asp60‐D1‐P1. The electrophoresis results are shown in Figure 18. The Native‐PAGE electrophoresis analysis results are shown in Figure 18A. The standard equation of mobility (*Y* = 5.6151x‐8.6915, *R* ^2^ = 0.9934) was obtained by drawing the standard curve of the logarithm of the molecular weight of the standard protein and mobility. As shown in Figure 18B, Asp60‐D1‐P1 presented two clear protein bands on the non‐denaturing electrophoresis gel. The molecular weights of Asp60‐D1‐P1 were estimated to be 44.5 and 130 kDa, respectively. Large molecular bands may be protein polymers of small molecular bands.

**FIGURE 18:**
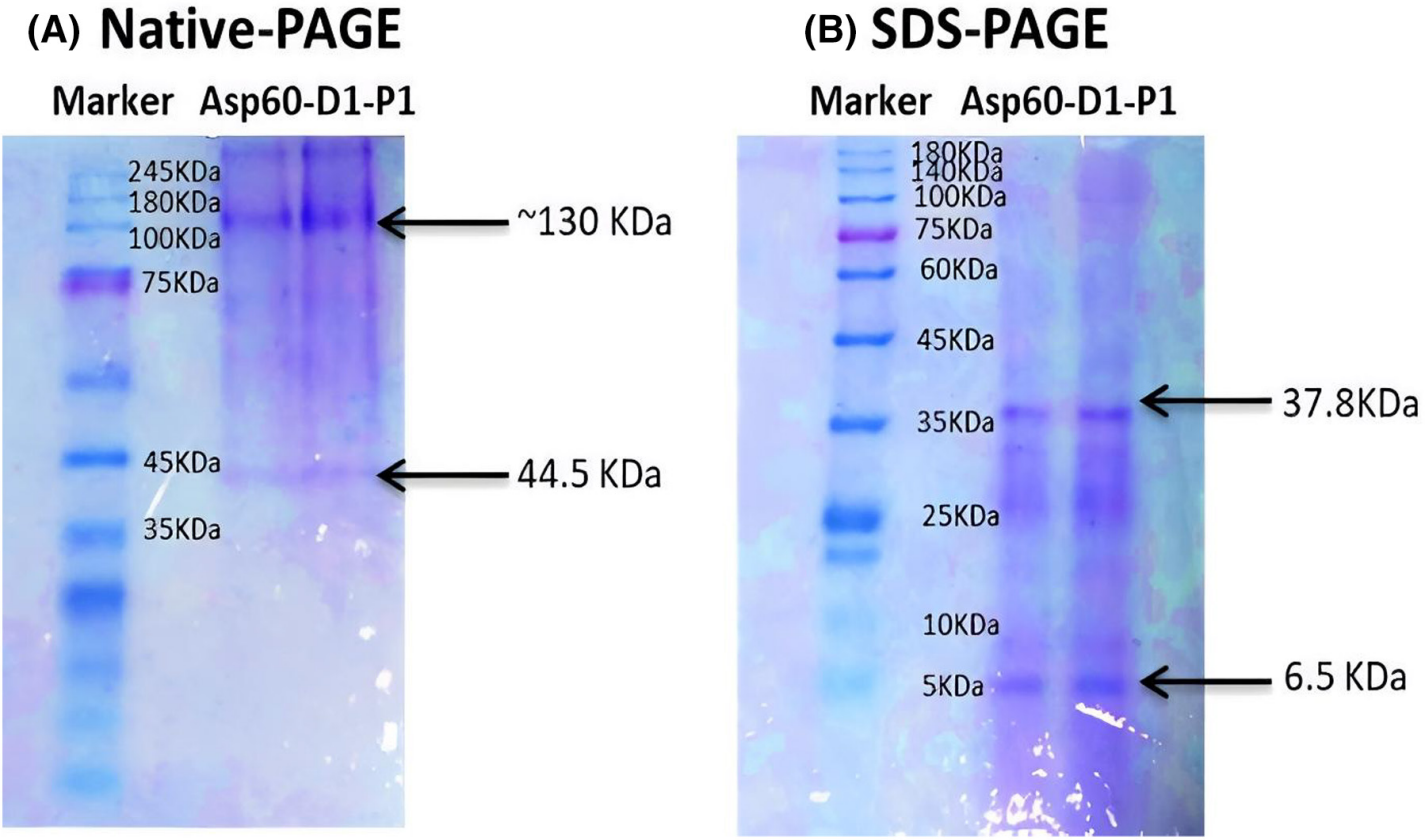
The PAGE pattern of Asp60‐D1‐P1 (A. Native‐PAGE, B. SDS‐PAGE).

Based on the results of SDS‐PAGE electrophoresis, the standard curve equation of protein molecule mobility was also obtained: *Y* = 2.9714x‐2.2665, *R* ^2^ = 0.9897. After Asp60‐D1‐P1 was denatured to release protein subunits, two clear peptide bands appeared on the electrophoresis gel (Figure 18B). According to their mobility, the molecular weights of Asp60‐D1‐P1 were 6.5 and 37.8 kDa, respectively.

By combining the results of non‐denaturing and denaturing electrophoresis, we inferred that Asp60‐P1‐D1 is a single‐component protease with a molecular weight of about 44.5 kDa, and its structure contains protein subunits with molecular weights of about 37.8 and 6.5 kDa, respectively. The purified Asp60‐P1‐D1 can exist in the form of protein trimers.

### 3.9 MS analysis

#### 3.9.1 Analysis of LC–MS results

Mr_44.5 and Mr_103 samples were detected by LC–MS; 104 (Figure 19A) and 126 (Figure 19B) peptide fragments with different abundance were detected by the base peak pattern within 60 min, and Uniprot_Perinereis_210_20190813 was searched by the Mascot2.2 software. In the FASTA database, Mr_44.5 and Mr_100 matched 21 proteomes, respectively (Table 10). By comparing the proteome results of the two samples, we found that catalase was only detected in Mr_44.5 and endoglucanase was only detected in Mr_130. By comparing the retention time and abundance of ionized peptide fragments of the two samples (Figure 19), we found that the digested fragments of the two samples were highly similar, and this result matched the conclusion in Table 10, which together indicated that Mr_44.5 and Mr_130 were identical, and this result matched the inference drawn in Section 3.8, i.e., the protein bands of Mr_130 were the multimers of the protein bands of Mr_44.5.

**FIGURE 19:**
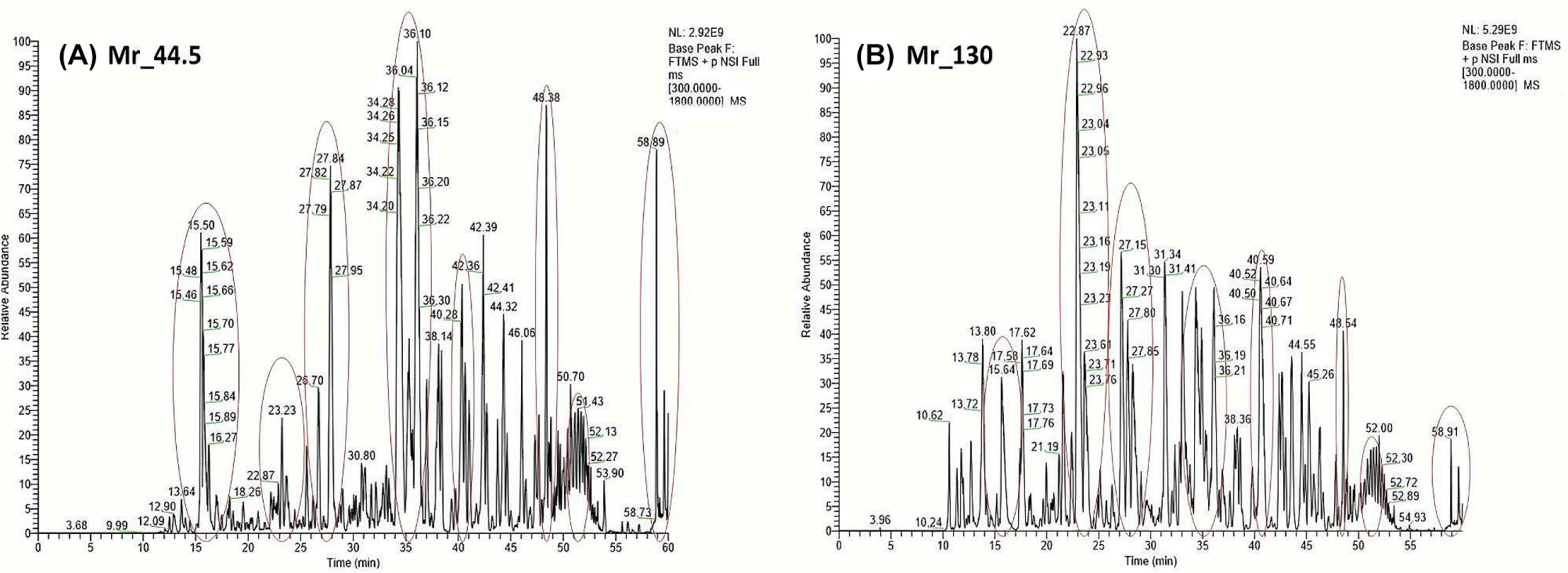
The base peak pattern of samples (A. Mr_44.5; B. Mr_130).

**TABLE 10:** The matched proteomic information of samples in the UniProt database.

Enzymatic peptide fragment proteomic information		Sample group	
UniProt library login number	Proteome name	Mr_44.5	Mr_130
tr|A0A0A7C0F7|A0A0A7C0F7_PERAI	Vitellogenin	1	1
tr|G5DBJ1|G5DBJ1_PERAI	Actin (Fragment)	1	1
tr|B6E649|B6E649_PERAI	Metalloprotein	1	1
tr|A0A076JSQ6|A0A076JSQ6_PERAI	Ferritin	1	1
sp|P04572|SCP_PERVT	Sarcoplasmic calcium‐binding protein	1	1
tr|A0A1B1HY43|A0A1B1HY43_PERAI	Endoglucanase	1	1
tr|Q9GN70|Q9GN70_PERVT	Troponin C	1	1
tr|F2Z7L1|F2Z7L1_9ANNE	Endoglucanase	1	0
tr|A0A0A7CAN8|A0A0A7CAN8_PERAI	Catalase	0	1
tr|A0A410RHA6|A0A410RHA6_9ANNE	Extracellular globin (Fragment)	1	1
tr|A0A410RHD0|A0A410RHD0_9ANNE	Extracellular globin (Fragment)	1	1
tr|Q7M3V2|Q7M3V2_PERAI	Hemoglobin, extracellular, linker chain L2 (Fragment)	1	1
tr|Q7M414|Q7M414_PERAI	Hemoglobin, extracellular, chain A2 (Fragment)	1	1
tr|Q7M415|Q7M415_PERAI	Hemoglobin, extracellular, chain A1 (Fragment)	1	1
tr|Q7M420|Q7M420_PERAI	Hemoglobin, extracellular, chain a (Fragment)	1	1
tr|B8Y626|B8Y626_PERAI	Fibrinolytic protease	1	1
tr|A0A096X912|A0A096X912_PERNU	Superoxide dismutase [Cu‐Zn]	1	1
tr|A0A096X937|A0A096X937_PERNU	Superoxide dismutase [Cu‐Zn]	1	1
tr|A0A410RG48|A0A410RG48_9ANNE	Hemoglobin linker chain (Fragment)	1	1
tr|E5LLA5|E5LLA5_PERAI	Heat shock protein 70s	1	1
tr|Q7M417|Q7M417_PERAI	Hemoglobin, extracellular, chain B (Fragment)	1	1
tr|Q7M423|Q7M423_PERAI	Hemoglobin, extracellular, chain b (Fragment)	1	1

*Note*: UniProt library, “1”: detected; “0”: not detected.

#### 3.9.2 Analysis of the LC–MS–MS results

The results of in vitro experiments showed that the Asp60‐D1‐P1 protease of *Perinereis aibuhitensis* had high fibrinolytic activity, and its electrophoretically purified bands were subjected to QE. The enzymatic peptide fragments of Mr_44.5 and Mr_130 were matched with the fibrinolytic protease proteome in the database: tr|B8Y626|B8Y626_PERAI Fibrinolytic protease OS = *Perinereis aibuhitensis*, OX = 126 650, PE = 2, and SV = 1. The results of the mass spectrometric secondary extraction of its peptides are shown in Figure 20.

**FIGURE 20:**
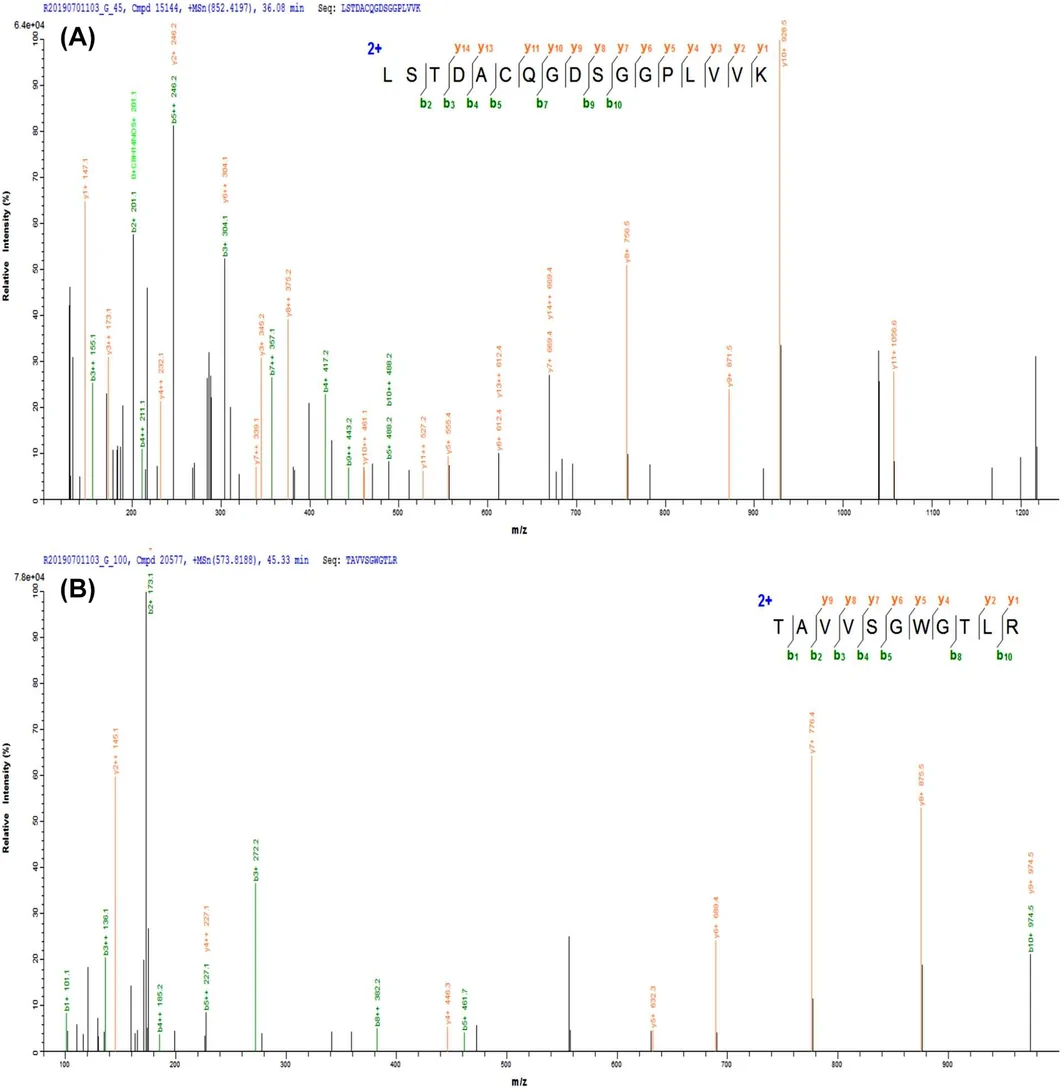
Partial interpretation of the LC‐MS‐MS spectrum of the fibrinolytic peptide in the sample. (A): R.LSTDACQGDSGGPLVVK.D; (B): R.TAVVSGWGTLR.S.

By conducting the LC–MS–MS assay and uniprot_Perinereis_210_20190813.fasta database matching, two peptide fragments of fibrillarin proteome were found in the samples, with amino acid sequences of R.LSTDACQGDSGGGPLVVK.D (Figure 20A) and R.TAVVSGWGTLR.S (Figure 20B), the theoretical molecular weight of the two peptides were 1703.8269 and 1146.6266 when the charge was 1, and the peptide scores were 91.86 and 40.41, respectively, which indicated that the peptide R.LSTDACQGDSGGPLVVK.D had a better match of the secondary maps. The FASTA package provides a comprehensive set of similarity searching programs, similar to those provided by the BLAST package, and some additional programs that are not provided by BLAST for searching with short peptides and oligonucleotides. FASTA provides very accurate statistical significance estimates and is more sensitive than BLAST when comparing DNA sequences. The amino acid sequence of a protein determines its tertiary/quaternary structure^25^ and, ultimately, its function. The two peptides were matched to the full amino acid sequence of fibrinolytic enzymes, and their secondary structures were predicted; the results are shown in Figure 21.

**FIGURE 21:**
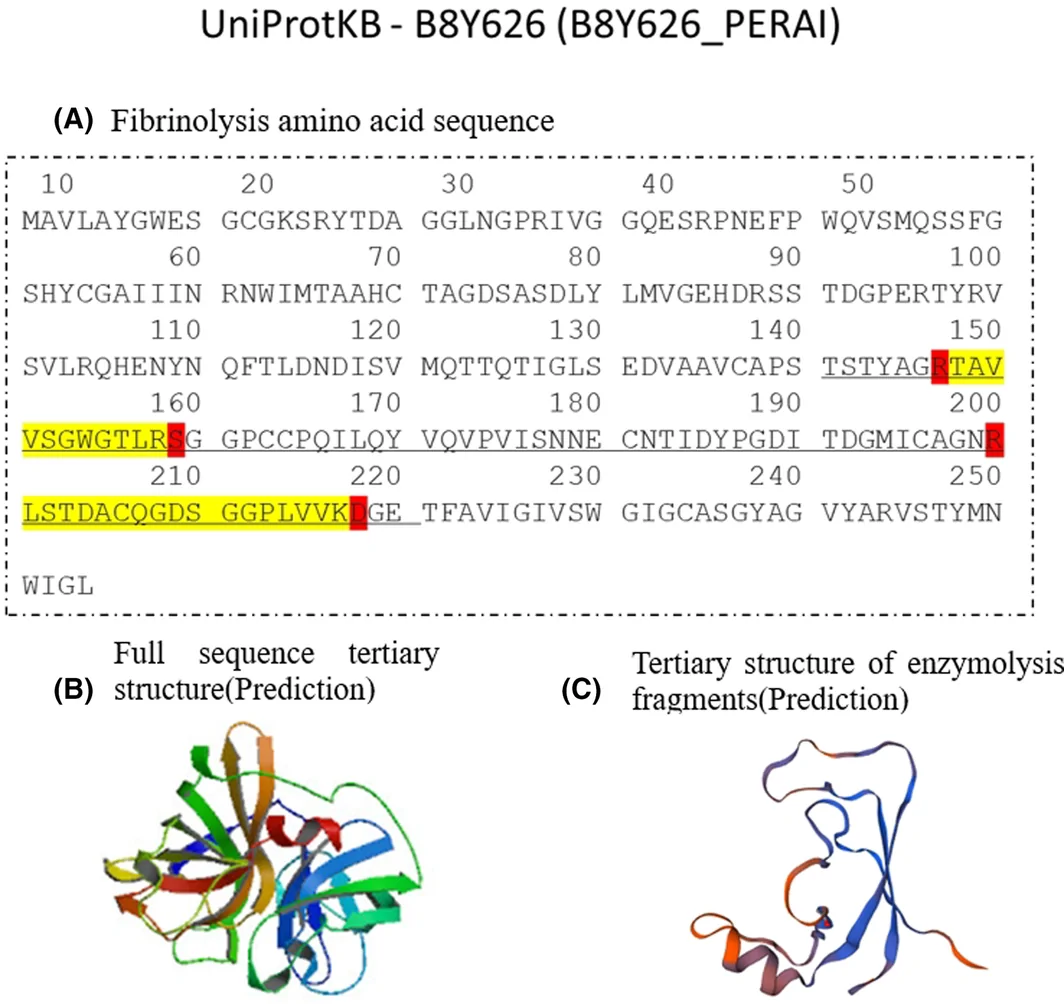
Second structure prediction of fibrinolytic enzyme and fibrinolytic‐matched peptide in Asp60‐D1‐P1. (A) Fibrinolysis amino acid sequence, (B) Full sequence tertiary structure (Prediction), and (C) Tertiary structure of enzymolysis fragments (Prediction).

## 4 CONCLUSION

### 4.1 The optimal purification processes

Gel filtration chromatography is a process used to separate proteins by adjusting the size of the pores in the filler according to the variations in the molecular weight of the proteins. During elution, the large molecular protein was blocked out of the pore, the volume at the lower end of the column was the smallest, and thus, it was eluted first; the small molecular protein eluted more slowly due to the larger volume. Finally, the peak was eluted. Anion exchange chromatography is a way to separate proteins based on the difference in charge. The side chain (−CH_2_CH_2_N^+^H(CH_3_CH_2_)_2_) of DEAE ion exchange resin carries a positive charge. During chromatography, the protein was positively charged. It was difficult for the protein to adsorb on the resin and thus it was eluted first. As the Cl^−^ concentration in the eluate increased, negatively charged proteins eluted in sequence according to the strength of their capacity to adsorb to the resin. Hydrophobic chromatography is a type of affinity chromatography. It is a way to separate proteins based on their differences in hydrophobicity. Hydrophobins bind to the hydrophobic groups (phenyl groups) on the side chains of gel particles at high salinity. As the salinity of the eluate decreased, the hydrophobin was eluted in sequence according to the strength of its hydrophobicity. Due to the high salinity of the non‐specific elution of hydrophobic chromatography, it may be unstable because of electrostatic effects. The structure of the protein was destroyed, which affected its activity.^26^

The experimental results showed that the best way to purify the fibrinolytic active protease of the bidentate silkworm is as follows: living silkworm → washing with sodium citrate and sodium chloride (hydrolysis of the shell, removal of silkworm toxin) → tissue homogenization → 40%–60% sulfuric acid Ammonium salting out → dialysis → freeze‐drying → anion exchange chromatography (DEAE‐Sepharose FF) → freeze‐drying → hydrophobic chromatography (Phenyl‐Sepharose FF) → freeze‐drying → *Perinereis aibuhitensis* Grub fibrinolytic active protease (Asp60‐D1‐P1).

### 4.2 Enzymatic properties

Polyacrylamide gel electrophoresis is a traditional method to analyze the purity and molecular weight of proteins. The protein sample was mixed with the loading buffer and heated to eliminate the difference in charge and molecular structure, and its molecular weight was estimated based on its mobility under constant voltage. This method was simple to operate and had a high accuracy rate. Asp60‐D1‐P1 was separated by Native‐PAGE to obtain two clear bands of the enzyme component, the molecular weights were 130 and 44.5 kDa, respectively. After denaturation treatment, the 130 kDa band disappeared, and two molecular weights appeared in the SDS‐PAGE film. For the bands 37.8 and 6.5 kDa, respectively, the sum of their molecular weights was close to the 44.5 kDa protein band in Native‐PAGE. Thus, the protein was inferred to be a double‐stranded fibrinolytic protease composed of two polypeptide chains. Regarding the 130 kDa band detected in Native‐PAGE, the molecular weight of the band was about three times that of the 45 kDa band. Based on this, it was inferred that the protein in the Asp60‐D1‐P1 sample freeze‐dried powder can exist in the form of a tri‐molecular polymer. The temperature stability results showed that Asp60‐D1‐P1 had good stability within the range of 20 to 60°C. Among them, the casein and fibrin hydrolyzing activity of the enzyme was the strongest at 40°C, and thus, the optimum temperature for this enzyme was judged at 40°C. The enzyme activity was lost when it exceeded 70°C. The pH stability results showed that the enzyme had good stability in a pH range of 6.0–11.0. Moreover, the overall activity of the enzyme in an alkaline environment was higher than that in an acidic environment. Therefore, the enzyme was considered to be an alkaline protease. The pH‐protease activity curve of the protein of Asp60‐D1‐P1 presented a double S curve. The first activity peak appeared at pH 8.0. After pH 9.0, the protease activity increased again, and the activity increased with an increase in alkalinity. By simulating the metal ion environment of blood, we determined the effect of metal ions on the activity of Asp60‐D1‐P1. The results showed that trace amounts of Na^+^, K^+^, Ca^2+^, Mg^2+^, and Fe^2+^ negligibly affected the enzyme activity, while the results of the Fe^3+^ comparison group showed that trace amounts of iron ions significantly inhibited the enzyme activity.

### 4.3 Mass spectrometry

The Asp60‐D1‐P1 protease peptide samples were obtained through Native‐PAGE purification and trypsin hydrolysis. The peptide samples were separated by liquid chromatography to obtain a single component solution directly in the mass spectrometer. After the peptides had been identified by mass spectrometry, they were searched using the Mascot 2.2 software. By comparing them with the uniprot_Perinereis_210_20190813.fasta database, the peptide fragments and proteomes were found to match the Mr_44.5 and Mr_130 samples, and the enzymatically digested fragment possessed about 95% similarity. Additionally, based on the electrophoresis result of 3.8, we found that there was a single protein component in Asp60‐D1‐P1, and the 130 kDa band in the two bands separated by Native‐PAGE was a trimer of the 44.5 kDa band. Mr_44.5 was matched with the enzymatically digested fragments from Mr_130 samples, R.LSTDACQGDSGGGPLVVK.D and R.TAVVSGGWGTLR.S. This match was found in the UniProt protein library of the fibrinolytic enzyme proteome. Combined with the results of the in vitro experiments in this study, we hypothesized that Asp60‐D1‐P1 is a kind of fibrinolytic enzyme in *Perinereis aibuhitensis* Grub.

In this study, fibrinolytic protease was isolated and purified from the white grub aibuhitensis, and its extraction technique was optimized. The properties of the enzyme were studied, and various experimental results showed that the crude protease activity of 40–60% salt‐out area was the highest (up to 467.53 U/mg). Using DEAE‐sepharose FF and Phenyl Sepharose 6FF to purify crude protein, the single enzyme Asp60‐D1‐P1 with the highest fibrinolytic activity was obtained. The optimum temperature was 40°C, and the optimum pH was 8.0. Combined with the results of Native‐PAGE and SDS‐PAGE, the molecular weight of Asp60‐D1‐P1 was found to be 44.5 kDa, which was composed of two subunits with molecular weights of 6.5 and 37.8 kDa, respectively. The method of purifying enzymes using MS combined with SDS‐PAGE and in vitro experiments is quite novel in this field. The available purification methods of plasminase are limited to the traditional protein purification methods in the biological field, which mainly have disadvantages such as slow chromatogram speed and insufficient accuracy to determine whether the isolated protein is the target protein or simply contains a small amount of the target protein. When combined with MS mass spectrometry, MS uses the precise quality information of peptide fragments, which is more specific and reliable, and greatly increases the reliability of the search results. In this study, the technique also decreased the uncertainty in the experiment related to the protease purification process; thus, it has practical applications.

## AUTHOR CONTRIBUTIONS

**Tuo Song:** Investigation (lead); validation (equal); writing – original draft (lead); writing – review and editing (lead). **Xinyuan Han:** Formal analysis (equal); investigation (equal); methodology (equal); writing – original draft (equal). **Tingting Jiang:** Methodology (equal); writing – review and editing (equal). **Xinyi Pu:** Investigation (equal); writing – review and editing (equal); Funding acquisition (supporting). **Mingjun Wei:** Resources (equal); writing – review and editing (equal). **Yuping Zhu:** Conceptualization (lead); investigation (equal); methodology (equal); validation (equal); writing – original draft (lead); writing – review and editing (lead). **Wenhui Wu:** Conceptualization (equal); funding acquisition (lead); resources (lead); writing – review and editing (equal).

## FUNDING INFORMATION

This research was supported by the Shanghai Frontiers Research Center of the Hadal Biosphere, the National Natural Science Foundation of China (Grant No. 82173731), and the Research Fund for International Young Scientists (Grant No. 81750110548).

## DISCLOSURES

The authors declare no conflicts of interest.

## ETHICS APPROVAL STATEMENT

The ethics governing the use and conduct of experiments on animals were strictly observed, and the experimental protocol was approved by the Shanghai Ocean University Committee on Medical Research Ethics.

## PATIENT CONSENT STATEMENT

Not applicable.

## Data Availability

The data that support the findings of this study are available on request from the corresponding author.
